# Thermotropic Ionic Liquid Crystals

**DOI:** 10.3390/ma4010206

**Published:** 2011-01-14

**Authors:** Kirill V. Axenov, Sabine Laschat

**Affiliations:** Institut für Organische Chemie, Universität Stuttgart, Pfaffenwaldring 55, D-70569 Stuttgart, Germany; E-Mails: sabine.laschat@oc.uni-stuttgart.de (S.L.); kirill.axenov@oc.uni-stuttgart.de (K.V.A.); Tel.: +49-(0)711-685-64565; Fax: +49-(0)711-685-64285

**Keywords:** liquid crystals, ionic thermotropic mesogens, metallomesogens

## Abstract

The last five years’ achievements in the synthesis and investigation of thermotropic ionic liquid crystals are reviewed. The present review describes the mesomorphic properties displayed by organic, as well as metal-containing ionic mesogens. In addition, a short overview on the ionic polymer and self-assembled liquid crystals is given. Potential and actual applications of ionic mesogens are also discussed.

## 1. Introduction

The liquid crystalline state is a state of matter in which orientational order is maintained, but, similarly to liquids, gases and amorphous solids, positional order in the molecular arrangement is lost [1]. Nowadays, materials forming liquid crystalline phases (*mesophases*) have found wide applications in the manufacturing of displays [2], spatial light modulators [3], optical connectors and switches [4], molecular sensors and detectors [5,6], and in many other topics [7]. A *thermotropic* mesophase is formed by heating a solid or cooling an isotropic liquid (or another mesophase), while *lyotropic* mesophases are prepared by dissolving an amphiphilic *mesogen* (a compound that displays liquid crystalline behavior) in suitable solvents, under appropriate conditions of concentration and temperature [1]. The conventional design of mesogenic molecules, exhibiting liquid crystalline (*mesomorphic*) properties, could be modified by an introduction of ionic groups, leading to ionic liquid crystals [8]. Actually, ionic liquid crystals are closely related to ionic liquids, which have attracted a growing interest as solvents with easily tunable physical and chemical properties [9]. Due to the presence of ionic units in the mesophase, the typical feature of ionic liquid crystals is ion conductivity, and this phenomenon can be used for the construction of materials with anisotropic electric current conductivity (see below) [10]. In general, ionic interactions tend to stabilize lamellar mesophases, due to an ion-ion stacking and electrostatic interactions [11]. This has already recently been observed for ionic liquids. Detailed small angle neutron scattering of *N*-alkyl*-N*-methylimidazolium-based hexafluorophosphate ionic liquids displayed the presence of local anisotropy in the bulk, isotropic and ionic liquid phases [12]. The strength and intensity of the diffraction peak correlated with the length of the alkyl substituent in the imidazolium cation. Therefore, it has been concluded that there is still no long-range molecular aggregation and local order (*ca.* 1-2 Å) results from increasing anisotropy of the long-chain-substituted amphiphilic imidazolium cation [12]. Ujiie and coworkers have obtained the first experimental evidence, showing a significant stabilization of the thermotropic ionic mesophase, compared with a conventional LC (liquid crystalline) molecular arrangement [13]. They observed a slight decrease of the melting points and, at the same time, strong increase of the clearing points of the azobenzene-derived ammonium mesogens over their neutral analogs [13]. A similar phenomenon was recently described for triphenylene-based ionic systems. Incorporation of imidazolium ion functionalities into the paraffinic side-chain termini of a triphenylene derivative resulted in the stabilization of the columnar mesophase (see below) [14]. Another unique feature of the liquid crystals, formed by ionic amphiphilic molecules, is their spontaneous homeotropic alignment on a glass surface. It has been claimed that this spontaneous self-organization of the ionic mesophase is caused by interactions between cationic head groups and the surface and by an arrangement of hydrophobic tails, thus creating a monodomain of similarly oriented molecules of the ionic mesogen [15].

**Scheme 1:**
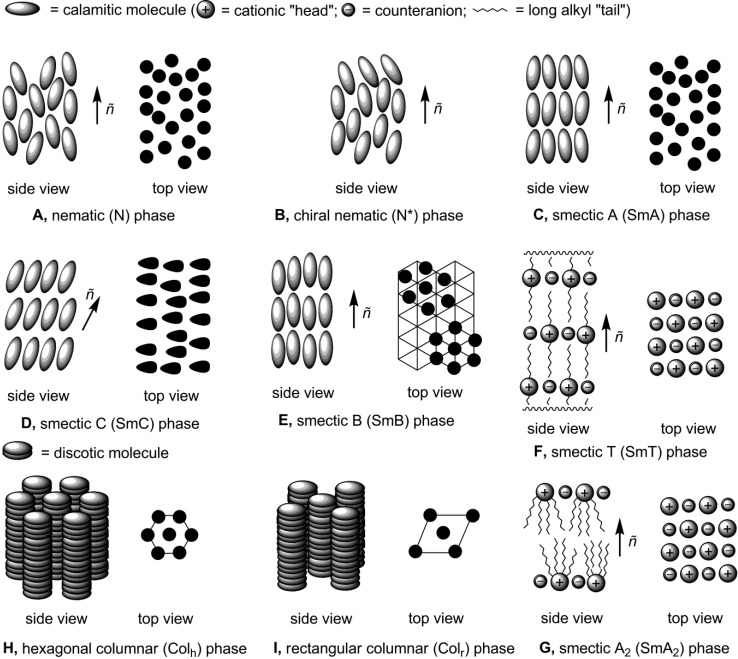


According to the type of order by which molecules are arranged in the liquid crystalline state, several mesophase types can be distinguished: *nematic* (N), *smectic* (Sm), *columnar* (Col) and *cubic* (Cub) [16,17]. A nematic (N) mesophase is usually formed by rod-like (*calamitic*) molecules. In the nematic type of molecular organization, molecules are arranged in random positional, but directionally correlated order. They are aligned in a general direction, defined by a unit vector *ñ*, the so-called director axis (**A**, Scheme 1). Chiral calamitic structures form chiral nematic (N*) mesophases, in which molecules are arranged in a helical manner (**B**, Scheme 1). In smectic order, calamitic molecules are organized in lamellar supramolecular assemblies and all oriented along the vector *ñ.* There are several types of smectic mesophases. In a smectic A arrangement (SmA), molecules are assembled in layers. In a layer, molecules are positionally random, but directionally ordered with their long axes normal to the plane of the layer (**C**, Scheme 1). In a smectic C phase (SmC), molecules have the same lamellar arrangement like in a SmA phase, but the vector *ñ* is tilted to the plane of a layer (**D**, Scheme 1) [16,17]. In an ordered smectic B (SmB) phase, there is short-range positional order within the layer. The neighbor molecules are arranged in six-fold bond-orientational order, which is lost within few intermolecular distances (**E**, Scheme 1).

In the conventional design, the ionic amphiphilic mesogenic molecule consists of a positively charged cationic head and long-chain hydrophobic alkyl substituent. The driving forces, leading to the formation and stabilization of the ionic mesophase, in a first approximation, are cation-cation repulsion, the Van-der Waals hydrophobic interactions between long alkyl tails and hydrogen bonding between anions and cations [18]. Microsegregation of incompatible units, aggregation of compatible units and the minimization of volume in bulk of ionic mesogene lead at suitable temperature conditions to appearance of a lamellar thermotropic ionic mesophase [11]. Due to a combination of these repulsive and attractive electrostatic forces, Van-der Waals hydrophobic interactions and hydrogen bonding contacts, unique smectic type molecular arrangements are found in ionic liquid crystals. A smectic T arrangement consists of specific order, exhibited by ammonium mesogens (see below) and characterized by tetragonal layers, separated by hydrophobic long alkyl chains (**F**, Scheme 1). A bilayered SmA_2_ mesophase is typical for phosphonium salts (see below). In a SmA_2_ phase, cations and anions are assembled in bilayers, separated by a double layer of alkyl chains (**G**, Scheme 1). The cubic phases (Cub) are molecular arrangements, having cubic symmetry (see below). 

*Discotic* molecules usually form columnar mesophases. In this supramolecular organization, molecules are stacked on top of each other into columns, which are arranged either in hexagonal Col_h_ or rectangular Col_r_ order (**H** and **I**, Scheme 1, respectively). The discotic molecules can also form nematic phases: a nematic D phase, with short molecular axes oriented in one preferential direction, and a columnar nematic phase (N_col_), in which molecules are stacked into columns, arranged in nematic order [11].

**Scheme 2:**
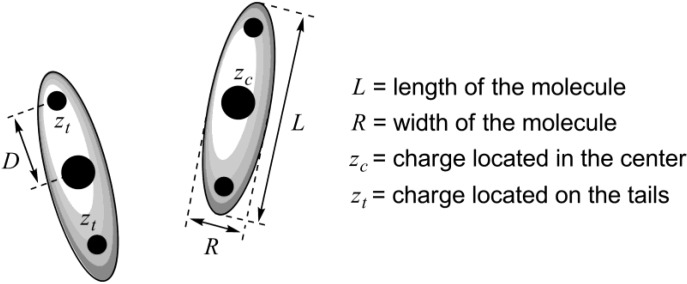


Until today, there is no conventional theory that could predict, starting from a known structure of a mesogenic ionic liquid, which type of mesophase this compound would form, and how stable this mesophase would be. Recently, employing density functional theory, a new theoretical approach has been developed, attempting to explain the influence of the anisotropic charge distribution on mesophase stability in ionic liquid crystals [19]. Ionic mesogenic molecules have been represented as ellipsoidal particles with length *L*, width *R* and charges located in the center or on the tails (with distance *D* from the center of the molecule, Scheme 2). Attractive interactions were taken into account in terms of the Gay-Berne pair potential. Finally, it has been concluded that nematic order should be stable for the molecules, in which the single charge is located in the center or two like charges are each positioned in a distance *D* = 1.4 *R* from the center (Scheme 2). Stability of the nematic phase could be improved with an increase in strength of electrostatic Coulomb interactions. When *D* = *R*, the phase with smectic A molecular arrangement is only stable. In certain cases, increasing the length of a charged particle could have a stabilizing effect on the smectic A phase. Moreover, it must lead to growth of the layer spacing in the smectic A phase. The developed methodology provided theoretical insight into the underlying mechanisms responsible for the formation of bulk liquid crystalline phases from ionic liquids [19].

A detailed review on ionic liquid crystals has been published by Binnemans in 2005 [11]. In this respect, our main goal for this contribution was to shed light upon further developments of the topic during the last five years. Despite such a relatively short period, a literature search revealed an enormous amount of publications dealing with ionic mesogens. In order to keep our review concise, we chose to focus here on the properties of thermotropic ionic mesogens, leaving out lyotropic ionic liquid crystals. Excellent reviews on the topic of lyotropic mesogens can be found elsewhere [20]. 

In our Schemes, we used conventional notation of thermotropic liquid crystalline properties. Phase symbol is followed by the upper temperature limit, as measured during the heating and not during the cooling cycle. For selected examples, temperatures of *enantiotropic* phase transitions are given. *Monotropic* transitions, which could be observed only upon cooling, appear in parenthesis. The isotropic liquid state is indicated with the symbol (I). The melting point refers to the temperature, when the crystalline phase melts into the mesophase (or directly into the isotropic liquid). The clearing point is the temperature of the transition between the mesophase and the isotropic liquid [1].

**Scheme 3:**
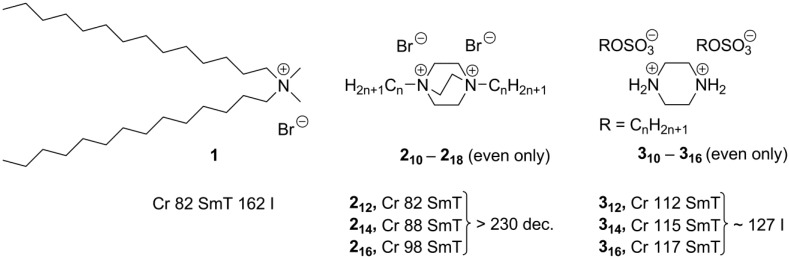


Due to the huge variety of mesogens considered in this review, it was rather difficult to create a unified numbering system. The basic structures are numbered through the text and Schemes by conventional arabic numbers. If an alkyl substituent with *n* carbon atoms is attached to this basic framework, it is displayed by coefficient right underline after the number of the structure. The counteranion is shown by its formula (with the negative charge omitted for clarity) in square brackets right after the number of the basic core (or *n* coefficient). For example, **2_12_[Br]** means that this compound has the basic structure **2** (Scheme 3), which bears C_12_H_25_-side-chains and has Br^–^ as a counteranion. For dendrimeric mesogens, the coefficient after the brackets displays a ratio between an amine dendrimeric core and an acidic second component in a self-assembled system (see Scheme 24).

## 2. Ammonium-Based Mesogens

Ammonium salts are simple amphiphilic molecules consisting of a substituted hydrophilic cationic nitrogen center and a hydrophobic hydrocarbon long-chain tail. Since synthesis of ammonium salts is simple and could easily be carried out from the corresponding amines via nucleophilic quaternization with alkyl halides [21,22], ammonium-based ILC (ionic liquid crystalline) compounds have been known for a long time. They form thermotropic or, in combination with a solvent, lyotropic mesophases. In addition, ammonium salts are widely used to build up a large variety of ionic self-assembled ordered structures (see below). In the absence of solvent, ammonium salts, in general, exhibit smectic ordered thermotropic mesophases [11]. One of the main reasons is that most of the popular ILCs are linear molecules consisting of a single ionic head group, connecting with one or multiple long aliphatic tails. It is generally accepted that the microsegregation of incompatible units, the aggregation of compatible units and the minimization of volume are the main driving forces that give rise to a general tendency for lamellar or columnar structures. 

Recently, for ionic ammonium compounds a new type of liquid crystalline order—smectic T phase—has been reported [23]. In a smectic T phase, ammonium head groups and halide counteranions are packed into tetragonal lattices separated from each other by randomly oriented long alkyl tails (**F**, Scheme1).Typically, the molecules with two long alkyl chain substituents exhibit such smectic T phases (**1**–**3**, Scheme 3) [23,24,25].

**Scheme 4:**
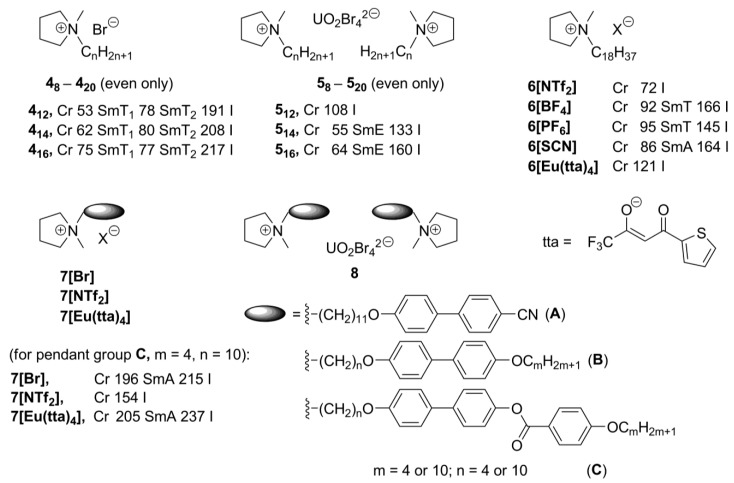


New pyrrolidinium-based mesogens **4_8_-4_20_** (Scheme 4) have been prepared by quaternization (Menschutkin reaction) of 1-methylpyrrolidine with long alkyl chain bromides [26,27]. The bromide anions can further be exchanged by noncoordinated or complex metal-containing counteranions. The pyrrolidinium compounds **4_8_-4_20_** show rich mesomorphism, depending on the length of the *N*-alkyl substitutuent and size of the counteranion. They melted into a LC phase in a range of 27–92 °C, and clearing points were observed between 171 and 267 °C. It was found that a minimum alkyl chain length of 11 carbon atoms is required for the pyrrolidinium bromide salts to exhibit mesomorphism. The tetrabromouranyl salts **5_8_-5_20_**, with a chain length of at least 14 carbon atoms, showed liquid crystalline SmE and SmA phases, while no liquid crystalline behavior was observed for the compounds **6[NTf_2_]** and **6[Eu(tta)_4_]**, containing NTf_2_^–^ (NTf_2_^–^ = N(SO_2_CF_3_)_2_^–^) or Eu(tta)_4_^–^ anions, respectively (Scheme 4) [26]. 

In the further improvement of the basic *N*-alkyl pyrrolidinium structure, mesogenic pendant biphenyl-derived groups were connected with the pyrrolidinium moiety via a flexible alkyl link (**7[Br]**, **7[NTf_2_]**, **7[Eu(tta)_4_]**, Scheme 4). Except the pyrrolidinium derivative **7[NTf_2_]**, bearing NTf_2_^–^ anions, these compounds feature a wide range of low and high ordered smectic phases (SmA, SmC as well as E, G, J, H, or K) [27]. The salts, which bear longer spacer and terminal alkyl substituent, showed lower melting points than their short-chain analogs. The europium-containing salts **7[Eu(tta)_4_]** were not liquid-crystalline. It has been stated that the Eu(tta)_4_^–^ anion is perhaps too bulky to be efficiently counterbalanced by the mesogenic units that were used [27]. However, by mixing the europium-containing salts with their bromide analogs **7[Br]**, luminescent liquid-crystalline mixtures could be obtained. The related pyrrolidinium systems have been recently investigated with the purpose to enhance electrochemical stability of an ionic liquid crystalline molecular arrangement. These new salts exhibited rich mesomorphism and formed mesophases that were stable over a wide range of temperatures [28].

**Scheme 5:**
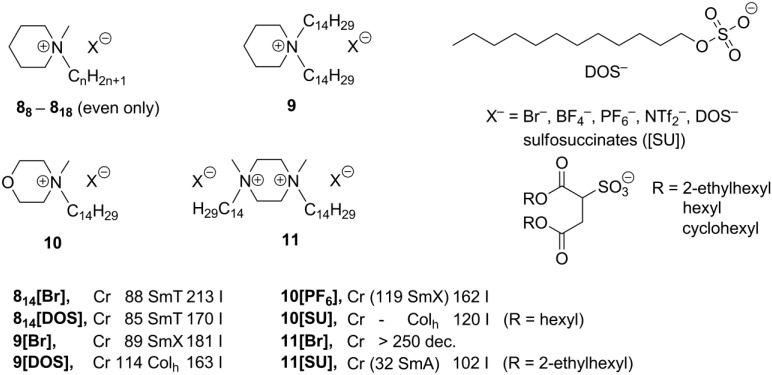


In order to explore whether ionic liquid crystals based on other aliphatic heterocycles could exhibit unusual mesophases, piperidinium, piperazinium, and morpholinium-based salts **8**–**11** (Scheme 5) were investigated [29]. It was also attempted to lower the melting points of the compounds to such an extent that they might exhibit mesophases at ambient temperatures. Consequently, sulfosuccinates, substituted with long alkyl chains, were combined with cationic ammonium mesogenic cores, apart from the more classical anions, like Br^–^, BF_4_^–^, PF_6_^–^, or NTf_2_^–^ (Scheme 5). Diverse mesophases have been observed for these compounds. The piperidinium salts **8[X]** and **9[X]** (X = Br^–^, BF_4_^–^ or PF_6_^–^), bearing one or two long alkyl groups (Scheme 5), feature SmT or SmE/SmT phases, while salts **8_14_[X]** and **9[X]** (X = DOS sulfate anion, Scheme 5) formed hexagonal columnar phases around 100 °C. In general, morpholinium-based structures behaved similarly and exhibited SmE and/or SmT phases (at higher temperatures less ordered SmA) [18]. All morpholinium-sulfosuccinate salts **10[SU]** (Scheme 5)showed hexagonal columnar phases already at room temperature and had clearing points between 120 and 147 °C. For the piperazinium-based compounds **11**, only those, bearing sulfosuccinate anions, displayed liquid crystalline properties and most of them formed smectic phases (SmA or SmE and SmT) at temperatures below 100 °C [29].

**Scheme 6:**
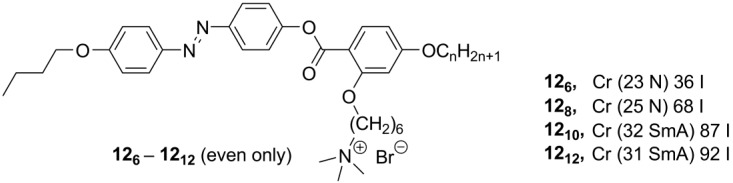


Recently, new mesogenic ammonium salts **12_6_-12_12_** (Scheme 6), containing a calamitic azobenzene core with lateral substituents, have been reported [30]. Very unusual for ammonium mesogens, the derivatives **12_6_** and **12_8_** displayed a nematic mesophase. In order to realize nematic ILCs, a series of branched (close to T-shaped) quaternary ammonium salts have been designed, by connecting an azobenzene unit with a benzoate link to a lateral ammonium bromide group (**12_6_-12_12_**, Scheme 6). Depending on the length of a terminal alkyl group, the compounds **12_6_-12_12_** formed nematic (n = 6, 8) or SmA (n = 10, 12) mesophases with melting points between 23 and 32 °C and clearing points in the range of 36–92 °C [30].

It can be concluded that in the last five years, the variety of ammonium-based mesogens has been expanded by involvement of new mesogenic groups—cyclic ammonium cations. The stability of mesophases, formed by ammonium molecules, is mainly depended on the lengths of *N*-alkyl substituents and used counterions. It should be noted that the highly polarized NTf_2_^–^ and metal-containing anions disfavor the formation of a mesophase. Conversely, stable smectic and columnar arrangements were obtained in the presence of bulky organic sulfates. Nematic mesophases have been observed for the mesogens constructed in a form of T-shape. Evidently, besides the counteranion and length of *N*-alkyl group, the position of a positively charged head group relative to a mesogenic tail is equally important for mesomorphic properties of an ionic mesogen (see also reference [19]), and this could be successfully used for the tuning of molecular order in an ionic mesophase.

## 3. Phosphonium-Based Mesogens

**Scheme 7:**
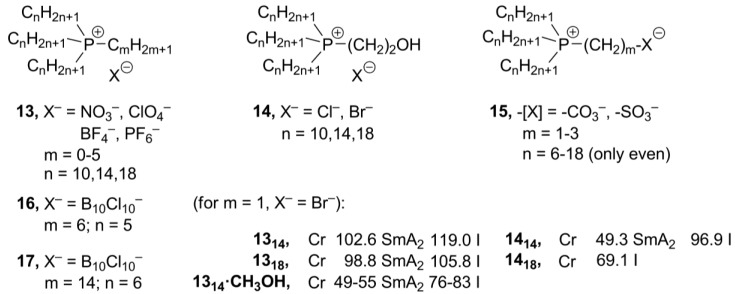


Due to the limited availability and increased chemical reactivity of alkylphosphines, the corresponding phosphonium salts find not so extensive application as ionic liquids, compared with their ammonium analogs [11]. Typically, phosphonium centers can be formed by quaternization reaction of trialkylphosphines with alkyl bromides. In order to avoid the presence of halides in the ionic product, alkyl sulfates or phosphates can be used as alkylating agents [31]. Although phosphines are less basic than the corresponding amines, their larger radii and more polarizable ionic pair make them more nucleophilic and, therefore, more reactive in alkylation reactions [32,33]. As it was already reviewed earlier [11], the phosphonium-based mesogens preferably form smectic A_2_ phases. Such SmA_2_ phases are composed of double layers of *n*-alkyl chains separated by ionic planes with the molecular long axes, which are orthogonal to the layer planes (as in a SmA phase). The A-type designation results from disorganized arrangements of the alkyl chains within a layer, although the positively charged head groups and their counterions are probably more regularly packed within ionic planes (**G**, Scheme 1) [34]. The most remarkable feature of the thermotropic phosphonium-based mesogens **13** (Scheme 7) is their ability to broaden their LC temperature range and to diminish their onset temperatures upon the addition of one or more equivalents of a protonic solvent, such as water or an alcohol [35]. It has been stated that noncovalent interactions between hydroxyl groups of a solvent and charged phosphorus centers induce mesomorphism and attenuate ion pairing [36,37]. Recently, new phosphonium mesogens **14**, containing β-hydroxyethenyl groups, have been prepared (Scheme 7) [38]. Due to intermolecular OH∙∙∙P^+^ interactions in these compounds, they displayed self-enhanced amphotropic behavior, similarly to it was earlier observed upon the addition of methanol to simple tetraalkylphosphonium mesogens **13**. In the absence of solvents, the phosphonium salts **13** showed melting points around 100 °C and clearing points between 106.2 and 114.0 °C, while phosphonium mesogens **14**, with embedded hydroxyl groups and the methanol solvates **13∙CH_3_OH** (m = 1, X^–^ = Cl^–^, Br^–^; Scheme 7), featured considerably lower melting points (at 43.5–85.0 °C; for **13∙CH_3_OH** 49.7–54.9 °C) and clearing points (73.4–99.0 °C; for **13∙CH_3_OH** 27.5–82.5 °C) [38]. In another approach, the phosphonium-“ate” zwitterionic structures **15** have been designed (Scheme 5), with an idea to weaken cation-anion contacts within an ionic layer and, at the same time, to establish new weak interactions between counteranions and “ate” groups in the phosphonium cations [39]. It was believed that this could help to maintain smectic lamellar order upon melting of the crystalline phase. However, none of the synthesized zwitterionic salts **15** showed liquid crystalline behavior. It has been proposed that the increase in disorder within lamellae of these salts frustrates melting into a liquid crystalline phase [39].

A number of new phosphonium salts **16** and **17** bearing perhalogenated carborane anions has been prepared (Scheme 7) [40]. Unfortunately, the authors did not report any mesomorphic properties of the new phosphonium products. It was only mentioned that the use of longer alkyl substituents resulted in a remarkable decrease of the melting points (**17**, m.p. 53 °C *vs.* **16**, m.p. 239 °C, respectively).

Phosphonium salts with long-chain alkyl substituents gave smectic liquid crystalline phases. Polar OH groups from the solvent or modified long alkyl chains enhance the stability of liquid crystalline order. It seems that the large phosphonium cation is easily accessible for the polar OH groups, placed in its close vicinity, and relatively strong hydrogen-bonding R_4_P^+^···HO contacts could be established, thus stabilizing a mesophase.

## 4. Imidazolium-Based Mesogens 

**Scheme 8:**
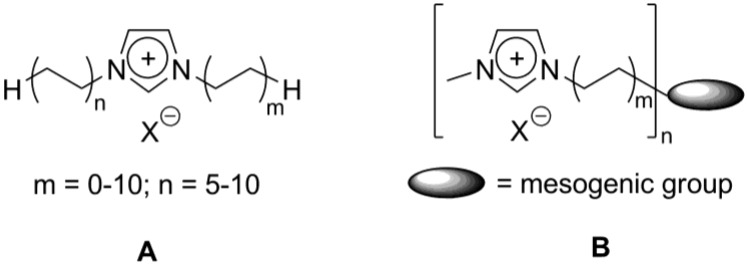


Most of the so far studied systems are imidazolium-derived ionic liquid crystals. This is connected with the extensive use of imidazolium salts for the synthesis of cheap and environmentally friendly ionic liquids [41]. Besides that, imidazolium compounds are easily accessible and stable precursors for the generation of late transition metal *N*-heterocyclic carbene complexes, employed for selective catalytic processes [42]. For imidazolium-based ionic liquids, the *in situ* formation of metal-carbene complexes has been attributed to be one of the key factors in the improvement of their catalytic behavior [43,44]. Reported to date liquid crystalline materials, based on imidazolium, can be classified into two types in terms of their molecular structure (Scheme 8) [11]. 

In the first type (**A**, Scheme 8), the imidazolium group acts as a mesogenic core, which is substituted by one or multiple long aliphatic tails. In most of such cases, alkyl substituted imidazolium salts exhibit smectic mesophases [41]. Here, the molecules are arranged into layers due to a combination of electrostatic interactions in the head group region and weaker van der Waals forces in the hydrophobic tails [12,46]. Due to a flexibility of the imidazolium core, even in the presence of large counteranions, the system is able to compensate in a smectic A rearrangement the difference between the cross-section of the tails and the ionic lattice area by the gathering of the ionic sublayer and by folding of the tails. In this situation, hydrophobic interactions between alkyl chains and hydrogen bonding interactions are important factors to induce and stabilize the mesophase [47]. For this type of imidazolium mesogens, the temperature range of the observed mesophase rapidly increases with the increasing alkyl chain length, although the alkyl chain length at which liquid crystalline mesophases appear depends on the anionic species [11,12,46,47]. Recently, by means of *ab initio* computer simulations, the structure and supramolecular interactions have been modeled for dialkyl-substituted imidazolium ionic liquids in different states of aggregation, from crystals to liquids and clusters. It has been concluded that a balance between Coulomb, van der Waals, and, sometimes, moderate hydrogen-bonding interactions, determines the macroscopic properties and behavior of imidazolium ionic liquids. This balance is crucial, when describing structures of reduced dimensionality, such as surfaces, interfaces, and clusters [48]. Small angle neutron scattering (SANS) methods have been applied for structural studies of the imidazolium ionic liquids, having alkyl chains with an intermediate length (**A**, m = 1; n = 4, 6, 8; X^–^ = PF_6_^–^, Scheme 8). They revealed structural features [49], which connect the close-packed cation-cation radial distribution observed for 1,3-dimethylimidazolium hexafluorophosphate, to the bilayer spacing of the long-chain liquid crystalline ionic liquids [12,47]. The model compounds (**A**, m = 1; n = 2, 4, 6; X^–^ = NTf_2_^–^, Scheme 8) were investigated by a combination of physical-chemical methods, such as X-ray diffraction, adiabatic calorimetric measurements and temperature-dependent IR spectroscopy, supported by quantum-chemical calculations. As a result, new approaches for the structural identification of an ionic liquid mesophase by the use of temperature-dependent IR spectroscopy have been developed [50].

Alternatively, the imidazolium group could be connected via a flexible linkage to a conventional liquid crystal mesogen on the tail ends (**B**, Scheme 8). In these types of imidazolium-based materials, the liquid crystalline properties originate from their strong amphiphilic character. The ionic interactions of the imidazolium groups stabilize both SmA and SmE phases. Stable columnar phases have been obtained for the compounds, where imidazolium groups have been attached on the tail ends of discotic molecules (see below).

**Scheme 9:**
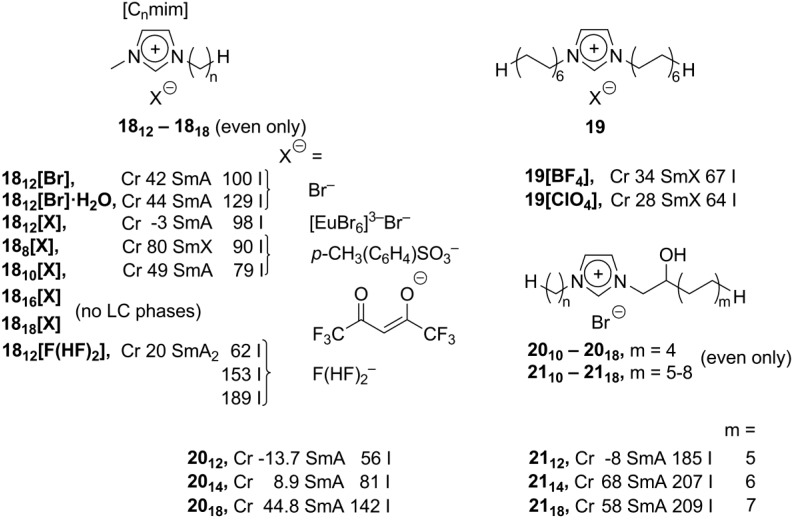


The type **A** imidazolium compounds could easily be modified with a variety of chemical methods, therefore synthesis and studies of mesomorphic behavior of new imidazolium salts still attract considerable attention. Recently, detailed studies of [C_16_mim]Cl (**A**, m = 1; n = 16; X^–^ = Cl^–^, Scheme 8) have been carried out to reveal a lamellar double-layer structure of the ABACAB type, which could be useful for further synthesis of mesoporous SiO_2_[51]. It is believed that, due to hydrogen bonding interactions, the presence of water could improve stability of a liquid crystalline phase formed by imidazolium mesogens [11]. As an example, [C_12_mim]Br·H_2_O hydrate (**18_12_[Br]·H_2_O**) showed a slightly increased temperature range for the smectic A mesophase, compared with the anhydrous analogs **18_12_[Br]** (Scheme 9) [52].

The basic [C_n_mim]^+^X^–^ structure could be simply modified by the use of new suitable counteranions X^−^. This approach provided a new series of easily accessible imidazolium mesogens, having wide spectra of interesting and applicable properties. With the complex large perhalogenated carborane anions, B_10_Cl_10_^2–^ and B_12_Cl_12_^2–^, imidazolium [C_n_mim] salts (n = 2, 16, 18; Scheme 9) exhibited smectic phases with high transition and clearing temperatures [40]. By an incorporation of Eu-containing counteranions, imidazolium-based luminescent mesophases **18[X]** (X = [EuBr_6_]^3–^Br^–^, Scheme 9) have been formed in the temperature range of −3 to 98 °C and determined as smectic phases [53]. It has been recently discovered that the [C_8_mim] and [C_10_mim] imidazolium salts **18_8_[X]** and **18_10_[X]** (X = tosylate anion, Scheme 9) generate lamellar mesophases being induced by shearing (or water traces) at room temperature. Conversely, bulk samples of these salts, investigated by DSC methods, display Cr-SmA(SmX) transitions between 50 and 80 °C [54]. Lately, utilizing the hexafluoroacetylacetonate counteranion, new hydrophobic ionic liquids **18[X]** (X = hexafluoroacetonate) have been synthesized and investigated (Scheme 9). While they did not show liquid crystalline behavior, it is worth to mention here that, remarkably, they were able to extract by tight complexation ions of late transition metals from water phase [55]. A series of new ionic mesogens [C_n_mim]^+^F(HF)_2_^−^ (**18[F(HF)_2_]**), bearing very polar fluorohydrogenate anion F(HF)_2_^−^, showed thermotropic smectic A phases in a broad temperature range (depending on the alkyl substituent, Scheme 9) and pronounced anisotropy in ionic conductivity [56].

In an alternative approach, the alkylimidazolium core could easily be modified by incorporation of the second long chain alkyl substituent [57] or small polar groups on the side-chain of the imidazolium mesogen [58]. These changes preserve the rod-like shape of the alkylimidazolium cation, and, consequently, keep smectic supramolecular order in the liquid crystalline state. At the same time, they allow tuning of the physico-chemical properties (temperature range, viscosity, *etc.*) of the ionic liquid crystal phase. The imidazolium salts **19[BF_4_]** and **19[ClO_4_]**, having two long dodecyl side-chain alkyl groups (Scheme 9), form smectic ordered mesophases at remarkably low temperatures of around 30 °C [57]. In the liquid crystalline state, these doubly substituted imidazolium salts exhibit non-Newtonian viscosity behavior (dependency of viscosity on shear rate), which is very unusual for ionic liquids and ionic liquid crystals. Above the liquid crystalline state, the viscosity of these compounds is independent of the shear rate. Consequently, non-Newtonian viscosity behavior of **19[BF_4_]** and **19[ClO_4_]** could be switched on and off, by keeping the temperature below or above of the phase transition point [57]. The presence of a small and polar hydroxyl group in the *β*-position to the imidazolium core (**20** and **21**, Scheme 9) leads to a considerable decrease of the Cr-LC transition temperature and to an expansion of the liquid crystalline temperature range. For a series of dialkylimidazolium salts **21** with two long alkyl substituents and one of them with an attached *β*-hydroxyl group (Scheme 9), an extremely wide mesophase temperature range has been observed (*ca.* 200 °C) [58]. Obviously, the hydrogen bonding interactions between hydroxyl substituents are weak enough to allow rearrangement of the molecules from solid to liquid crystalline state already at ambient or subambient temperatures. At the same time, these OH···OH contacts are strong enough to keep organized hydrophobic side-chains in layered smectic liquid crystalline order over a wide temperature interval.

**Scheme 10:**
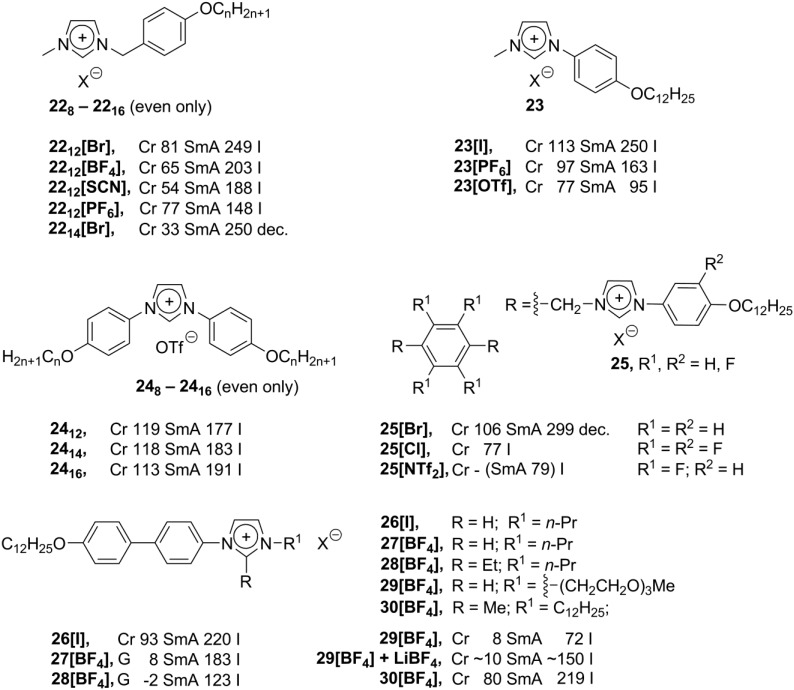


When the imidazolium cation is connected to a rigid (usually aromatic) mesogenic group, liquid crystalline properties of the ionic salt are defined by the structure of the rigid mesogenic skeleton (**B**, Scheme 8). Depending on the shape of mesogenic cores, imidazolium liquid crystals exhibit a huge variety of mesophases, from smectic to highly ordered columnar or even cubic phases. Calamitic benzyl groups, bearing long-chain alkoxy substituents in the *p*-position, could be simply connected with the imidazolium core by the reaction of corresponding benzyl bromides with imidazole. The bromide anion could further be exchanged by an anion metathesis reaction, which gave a series of benzylimidazolium salts **22** (Scheme 10) [59]. These compounds displayed low Cr-LC transition temperatures and high clearing temperatures. Small-sized anions stabilize the liquid crystalline phase (stabilization order: Br^−^ > BF_4_^−^ > SCN^−^ > PF_6_^−^). Lately, this series of imidazolium salts has been expanded by synthesis of new members with variable chain length of the *p*-alkoxy substitutuent in the benzyl ring (**22_10_-22_16_**, Scheme 10). All these structures **22_8_-22_16_** exhibited smectic A order in the liquid crystalline state [60]. 

Due to flexibility of the benzyl substituents in **22_8_-22_16_**, such benzylimidazolium structures possess a lot of conformational freedom. In order to create the rigid calamitic imidazolium molecules, the imidazolium cation and the aromatic mesogenic group should be connected directly. Unfortunately, aromatic halogenides are much less reactive towards nucleophiles than their aliphatic analogs; therefore, phenyl substituted imidazolium salts are barely accessible. Recently, based on transition metal-catalyzed coupling reactions, synthesis of new phenylimidazolium structures has been developed. Long-chain alkoxyphenyl-*p*-iodides have been reacted with imidazole under harsh conditions (Cu(II)NaY as catalyst, 72 h, 180 °C). After treatment of the products with MeI, new phenylimidazolium mesogens **23** (Scheme 10) have been obtained [61]. These compounds show typical smectic A phases, and it has been found that in the presence of highly polarized anions the clearing point and stability of the mesophase remarkably decrease. In another synthetic approach, the diphenylsubstituted imidazolium core **24** (Scheme 10) has been constructed from corresponding aromatic glyoxaldiimines. Their reaction with chlormethylpivaloate and AgOTf (AgOTf = AgOSO_2_CF_3_) afforded structures **24** with a high yield and purity [62,63]. The original compound **24_12_** showed smectic A molecular order and quite a high Cr-SmA transition temperature (119 °C) [62]. An increase in the length of the alkoxy substituent led to an increase of the clearing temperature (**24_14_** and **24_16_**, Scheme 10). The measured charger carrier mobility in the liquid crystalline state was in the range of 10^−4^ cm^2^/(V·s) [63].

Recently, synthesis and liquid crystalline properties of the phenylimidazolium salts **25** have been reported, where two imidazolium moieties were connected via the -CH_2_- bridges to the fluorinated aromatic core (Scheme 10). These compounds were synthesized with the purpose to study the influence of the fluorine groups on liquid crystalline properties. It has been recognized that four factors are important for liquid crystalline characteristics: the length of the alkoxy substituent, the type of the counteranion, and the position and number of the fluorine groups [64]. The complex multisubstituted imidazolium mesogens **26**–**30** (Scheme 10) have been synthesized via a Cu-mediated coupling between 2-alkyl-substituted imidazoles and a series of *p*-alkoxybiphenylbromides, *p*-alkoxyphenylbromides or *p*-alkoxybenzoatophenylbromides. On the other side of the imidazolium unit (in 3-*N* position), various substituents, including chiral branched systems, have been introduced [65]. Complex mesomorphic behavior of these compounds has been investigated. It has been found for the BF_4_^–^ anion-derived salts **27[BF_4_]** and **28[BF_4_]** that, compared with other analogs (**26[BF_4_]**, for example), the melting points decrease (Scheme 10). Moreover, in the presence of 1.0 eq. of LiBF_4_, the mesophase temperature range of salts **29** can be expanded, and smectic liquid crystalline order can be observed in the whole spectrum of temperatures from 0 °C to 150 °C. The compounds with larger cores display more stable SmA mesophases, while branching (either in a racemic or chiral form) in the alkyl substituent has a minimal influence on phase behavior. Short alkyl substituents enhance the formation of a SmA_2_ phase, and large lateral substituents destabilize the mesophase [65].

**Scheme 11:**
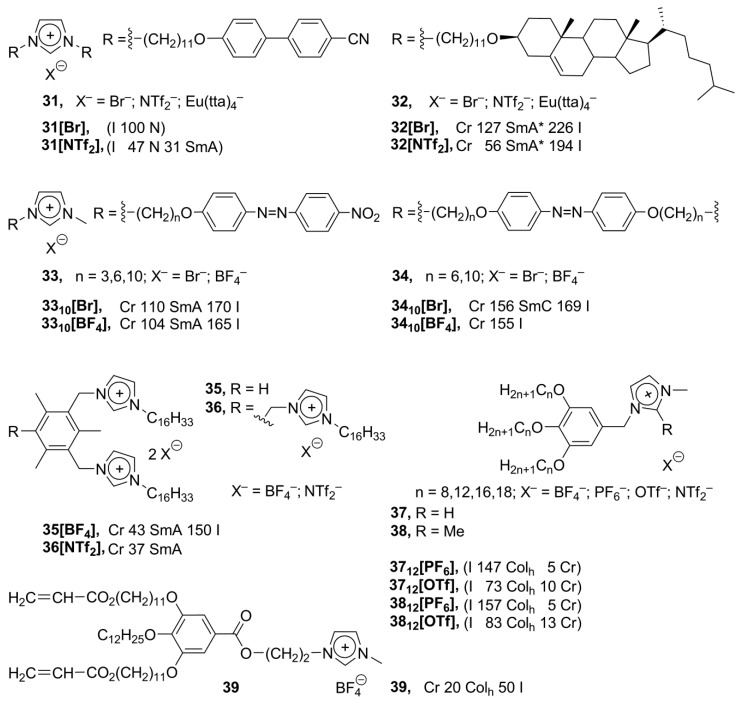


Despite the number of known synthetic methods employed to connect an aromatic mesogenic group directly with an imidazolium moiety, these structures are still not easy to access and modify. Simultaneously with synthesis and studies of the rigid-core imidazolium-based mesogens, a lot of attention was given to the preparation of complex flexible structures, where a mesogenic group was connected with the imidazolium core vi a along-chain flexible linker. The incorporation of the linker between an imidazolium and mesogenic group was provided using simple synthetic transformations, such as esterification or Williamson reaction. In such a way, new biphenyl-based **31** and cholesteric **32** imidazolium salts have been prepared (Scheme 11). Unexpectedly, along with the anticipated appearance of chiral SmA* phases for **32**, the formation of nematic phases has been observed upon cooling of an isotropic liquid of **31** [66]. In a similar approach, the azobenzene mesogens **33**, **34** have been connected with the imidazolium core via a long flexible alkoxy bridge (Scheme 11) [67,68]. The special feature that makes the azobenzene moiety particularly attractive is that the materials containing azobenzene chromophores undergo photoinduced modification of their absorbtion characteristics through reversible *trans*-*cis* isomerization by irradiation with the linearly polarized light [67]. The monocationic imidazolium salts **33** reveal smectic A liquid crystalline phases with the Cr–SmA transition points over 100 °C and a narrow temperature range of an LC phase (20–60 °C) [67]. The dicationic imidazolium compounds **34** formed ordered monolayered smectic C phases [68].

Bis- and triscationic imidazolium salts **35** and **36** (Scheme 11), bearing long-chain hexadecyl substituents, have been synthesized based on a mesitylene spacer [70]. These compounds showed low melting temperatures and formed smectic A phases in a wide temperature range. The salts with NTf_2_^–^anions tend to supercool before crystallization [70]. Recently, it has been shown that imidazolium-based discotic alkyl-substituted polyphenoles can form complex highly ordered hexagonal columnar phases. Being aligned on a surface within an enantiotropic columnar phase, these structures displayed anisotropic electric current conductivity [10]. In order to improve the conductivity of these compounds, new imidazolium derived polyphenol ethers **37** have been synthesized. Among a series of salts **37** (Scheme 11), it has been observed that the melting points show an increasing trend as the chain length increases. The counteranions in the salts **37** and **38** have order of the stabilization effect: BF_4_^−^ > PF_6_^−^ > OTf^−^ > NTf_2_^−^, typical for imidazolium mesogens [71]. The aligned and self-ordered on a surface hexagonal columnar organization with formed ionic channels can be fixed in **39** by a polymerization of the acrylate-derived alkoxysubstituents. As a result, stable 1D ion-conductive polymeric films are formed with enhanced ion-conductive properties [72].

**Scheme 12:**
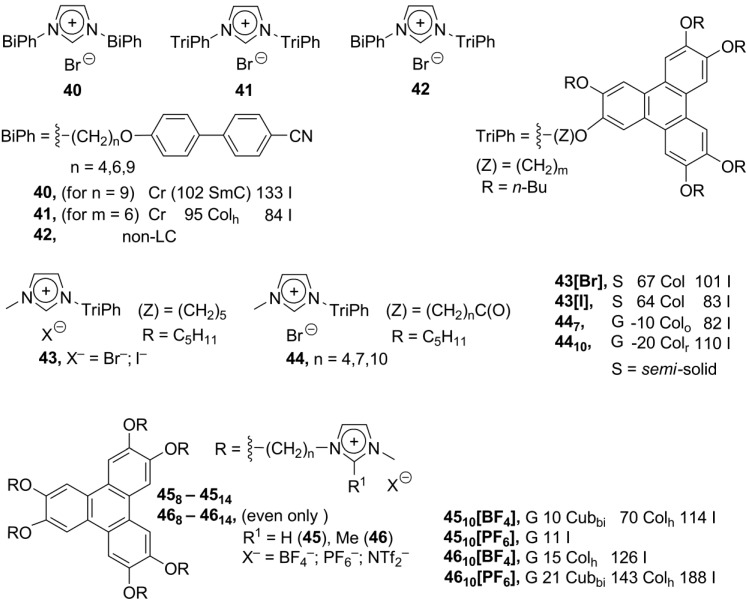


Besides polyphenol ethers, the well-known triphenylene group was used as a discotic mesogenic core for imidazolium ionic liquid crystals. With a series of biphenol-based calamitic and triphenylene discotic substituents, the calamitic-calamitic, discotic-discotic and calamitic-discotic imidazolium-based structures **40**–**42** have been synthesized by a microwave-assisted technique [73]. A combination of substituents, connected to the imidazolium cation, defines liquid crystalline properties of the investigated molecules. The calamitic-calamitic structures **40** exhibited tilted smectic C phases, while the discotic-discotic molecules **41** were organized into rectangular columnar phases (with the calamitic-discotic combination **42** being non-liquid crystalline) (Scheme 12).

Originally, the ether link (-O-) is typically used to connect the alkylimidazolium and triphenylene core (**43**, Scheme 12) [74,75]. Alternatively, the discotic imidazolium salts **44** have been obtained, where imidazolium and triphenylene moieties were bound via the ester spacer (-C(O)-O-) [76]. The mesogens **43** exhibited subambient Cr-Col transition temperatures, but narrow temperature windows for liquid crystalline columnar phases; while for the related compounds **44**, the lower limit of the liquid crystalline phase extended to −20 °C [74,75]. Lately, the multisubstituted triphenylene ionic mesogens **45** and **46**, bearing six charged imidazolium groups, have been designed (Scheme 12). In a comparison to the parent triphenylene and neutral imidazole-triphenylene analog, the compounds **45** and **46** exhibited a liquid crystalline phase with increased stability. With the externally added imidazolium salt [C_6_mim][BF_4_], the columnar phase is maintained over the temperature range from 4 °C to 117 °C. Obviously, ionic interactions within the mesophase stabilize the columnar assembly of the triphenylene moieties [14]. In more detailed synchrotron studies, it was realized that the optically “isotropic” phase, formed upon cooling of the parent columnar liquid crystals of **45** and **46** is in reality not amorphous but a mesophase with Cub_bi_ geometry. Columnar triphenylene arrays within cubic geometry are expected to enhance electric conductivity of the liquid crystalline substrate. It is believed that the bicontinuous cubic (Cub_bi_) geometry contains a three-dimensionally interconnected network of π-electron channels [77].

**Scheme 13:**
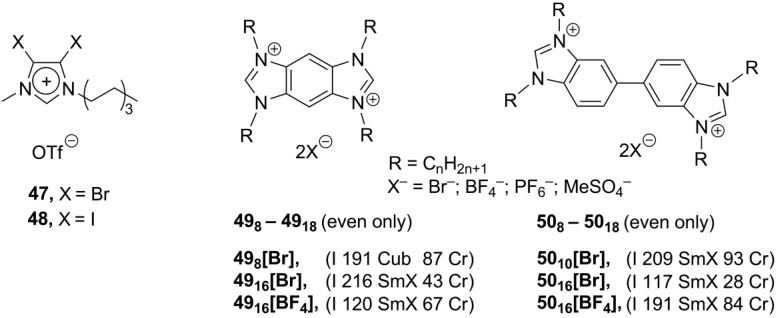


An additional way to modify the imidazolium core is to use related heterocyclic systems instead. In one approach, the halogenide substituents were introduced into an imidazolium ring. It was believed that they might stabilize the mesophase by a “halogen bonding effect”, interactions between an antibonding orbital of C-Hal groups as an acceptor and a Lewis base as an electron pair donor [78]. In the solid crystalline state, the compounds **47** and **48** (Scheme 13) feature a relatively short connection (around 2.9 Å) between the halogen imidazolium substituents and oxygen atoms of triflate counteranions. Unfortunately, none of the salts **47** and **48** revealed any of liquid crystalline properties. 

The extended aromatic systems can theoretically stabilize a mesophase by enhanced ionic and π-π type interactions [79]. With this purpose, the benzobis- **49** and bibenzimidazolium **50** systems (Scheme 13) have been constructed [80]. The compounds exhibited high thermal stability and form smectic or cubic liquid crystalline phases in a wide range of temperatures (*ca.* 34–220 °C). Mesophase stability of salts **49** rises with an increase in the length of the *N*-alkyl substituent up to C_15_, after which stability decreases, probably, due to plasticization effects. This trend was not observed for the bibenzimidazolium derivatives **50**. It seems that a free rotation in the dicationic core of **50** may disturb the liquid crystalline organization [80].

The cationic imidazolium core can be easily synthesized and further modified by simple chemical transformations. This ease of access places the imidazolium structures as one of the most applicable mesogenic groups so far, used for preparation of ionic liquid crystals. The mesomorphic characteristics of the imidazolium-based systems can be easily tuned by simple modifications of the substitution pattern around the imidazolium core. This led to a huge family of imidazolium ionic mesogens, which display diverse liquid crystalline phases, from smectic (alkylimidazolium salts) to highly ordered organization with molecular arrangements of columnar or cubic geometries (benzyl- or triphenylene-imidazolium mesogens). The highly ordered phases potentially could be used as anisotropic ion conductors (see below). Small, highly polarized anions, due to strong electrostatic and hydrogen bonding cation-anion interactions, display a stabilizing effect on the mesophase, while large “soft” anions, like SCN^–^ or I^–^, or “noncoordinating” anions, like PF_6_, rather destabilize liquid crystalline order. Together with a suitable counteranion, the presence of polar OH groups, even from traces of water, provide stable ionic mesophases, similarly to that observed for phosphonium mesogens (see above). From this point of view, the incorporation of –OH groups in the vicinity of an imidazolium core is a very promising synthetic strategy to build mesophases, stable over wide temperature intervals.

## 5. Pyridinium-Based Mesogens

**Scheme 14:**
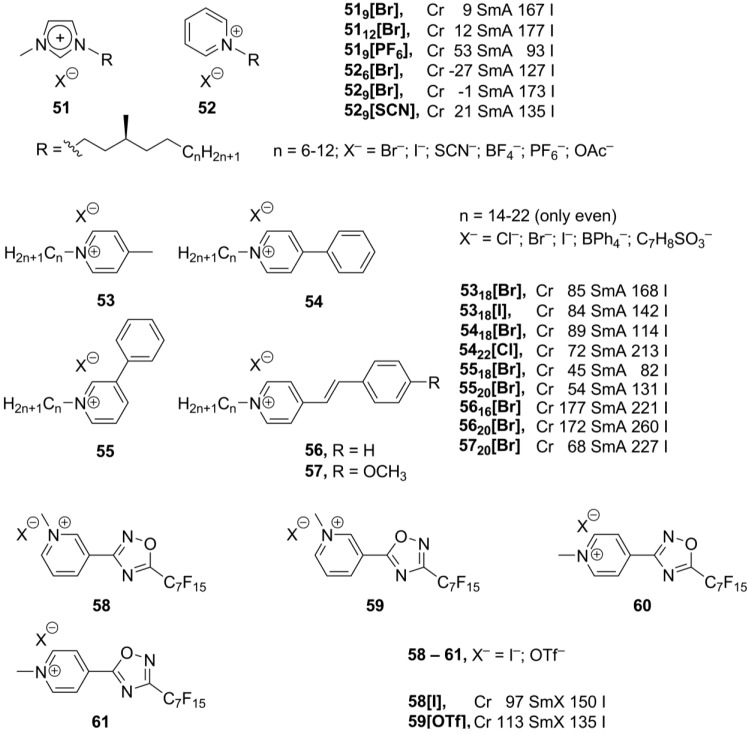


Pyridinium-derived mesogens exhibit properties similar to the related imidazolium mesogens. The driving forces for the formation of pyridinium and imidazolium ionic liquid crystals are the same: hydrophobic interactions of the long alkyl substituents and ionic, dipole-dipole, cation-π interactions as well as π-π stacking of the aromatic cationic core groups. Consequently, in most of cases, smectic liquid crystalline phases are expected for pyridinium salts [11]. Among organic cations, mesomorphism of pyridinium salts has been known since 1938 [81]. Later, it has been demonstrated that *N*-methylation (or more generally *N*-alkylation) is necessary for these salts to show mesomorphic behavior [82,83]. Recently, new imidazolium **51** and pyridinium salts **52** (Scheme 14), bearing *N*-alkyl substituents with a chiral center in the position four to the nitrogen atom, have been synthesized and investigated [84]. Both series, imidazolium and pyridinium salts, form smectic A phases and exhibit similar trends in the mesophase stabilization effect of alkyl groups and counteranions. Mesophase stability was observed to decrease in order, for **51**: Br^−^ > OAc^−^ > I^−^ > BF_4_^−^ > SCN^−^ > PF_6_^−^; for **52**: Br^−^ > OAc^−^ > BF_4_^−^ > I^−^ > SCN^−^. However, compared with their imidazolium analogs **51**, thepyridinium derivatives **52** have much lower solid-LC transition temperatures and clearing points [84]. 

Besides the alkyl long chains, other mesogenic groups have been employed for the design of new pyridinium-based mesogens (**53**–**57**, Scheme 14). It has been observed that, although the length of the *N*-alkyl group is important for stability of the mesophase, in general, the incorporation of a phenyl group is destabilizing the liquid crystalline state. The mesophase temperature range for the compounds **54_18_[Br]** and **55_18_[Br]** has been found to be much narrower than that recorded for the *p*-methylpyridinium analogs **53_18_[Br]** and **53_18_[I]** (Scheme 14) [85]. In the stilbazolium-based derivatives **57**, the presence of a donor methoxy group in the 4'-position of the aromatic system in the combination with the electron accepting pyridinium ring induces a large dipole moment in the molecule. This leads to a stabilization of a liquid crystalline phase (Scheme 14). All compounds **53**–**57** exhibit smectic A phases, with the head-to-tail arrangement of molecules within a layer. Counteranions are localized between positively charged pyridinium rings [85].

In a similar approach, the pyridinium core was modified by a connection with the 1,2,4-oxodiazole ring (**58**–**61**, Scheme 14). In order to maintain stability of a mesophase, long linear perfluorinated substituents have been attached to the mesogenic core in **58**–**61** [86]. It has previously been shown that the presence of a perfluorinated group in an appropriate position improves liquid crystalline properties [87]. Moreover, the linear substituents have better stabilizing effect over branched ones [88]. The 3’-substituted derivatives **58** and **59** displayed liquid crystalline properties, though in a narrow temperature range, while the 4’-oxodiazole salts **60** and **61** melt directly from solid state into isotropic liquids. To explain such different behavior of the 3’- and 4’-derivatives **58**–**61**, it has been proposed that the positive charge is delocalized over the whole cationic aromatic pyridinium-oxadiazole system, thus weakening cation/anion electrostatic interactions. The formation of smectic order in a liquid crystalline phase was established. In the solid crystalline phase, the hydrophobic perfluorinated tails are packed in extended bilayers in an end-to-end fashion [86].

**Scheme 15:**
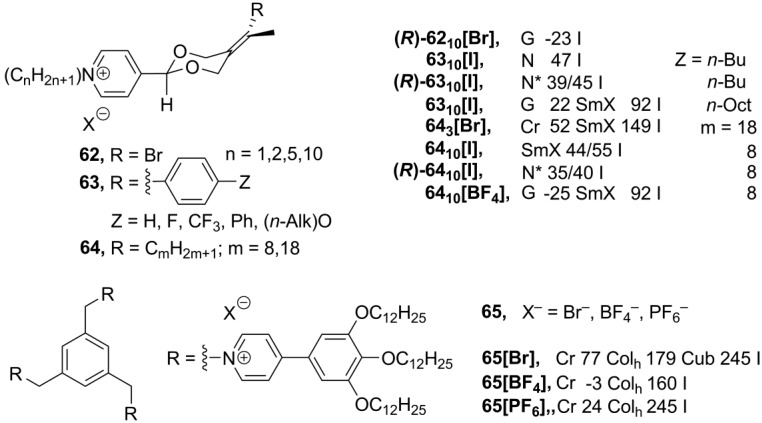


Recently, new ionic derivatives **62**–**64** (Scheme 15) with an axial chirality were reported [89]. Most of the compounds **62**–**64** exhibited different liquid crystalline phases, depending on the structure of the rod-like cation and the nature of counteranion. In these salts, two long-chain substituents in the cation are required to maintain a stable mesophase. Cholesteric (N*) phases have been observed for the enantiomerically pure salts, while racemic mixtures formed nematic or smectic achiral phases [89].

The imidazolium frameworks, based on discotic mesogenic cores, form extensive networks consisting of ionic molecules organized in a supramolecular columnar arrangement [11,14,73,74,75,76,77]. Similar ionic liquid columnar networks have been obtained from discotic pyridinium-derived structures **65** (Scheme 15). These tripodal discotic structures exhibit rectangular or hexagonal columnar liquid crystalline phases over wide temperature ranges, including room temperature. At higher temperatures, compound **65[Br]** forms micellar cubic phase, where each micelle is assumed to contain approximately 16 clustered molecules on average. Perhaps, the small-sized counteranions allow closer packing of ionic pyridinium cores and, consequently, favor the formation of a cubic phase over a columnar rearrangement [90].

The pyridinium-based mesogens display mesomorphic properties, similar to related imidazolium salts. Consequently, pyridinium-based structures can be used in practical applications as alternatives to imidazolium analogs, in particular for the fabrication of anisotrpic ion conductors. Stability of a mesophase is also depenent on the counteranions and the substitution pattern around the pyridinium core. Pyridine is a chemically stable structure, providing on the other side a reactive nitrogen center, which is easily accessible for synthetic transformations into a substituted mesogenic cation. Together with the substituent on the nitrogen center, the *p*-position at pyridine ring is also important to retain stability of a liquid crystalline phase. Placed in the *p*-position, donor alkyl (methylpyridinium), substituted donor aryl (structure **65**) or flexible vinylphenyl (*p*-stilbazolium) groups provide a positive stabilizing effect (compared with *p*-phenylpyridinium-derived mesogens).

## 6. Miscellaneous Organic Ionic Mesogens

**Scheme 16:**
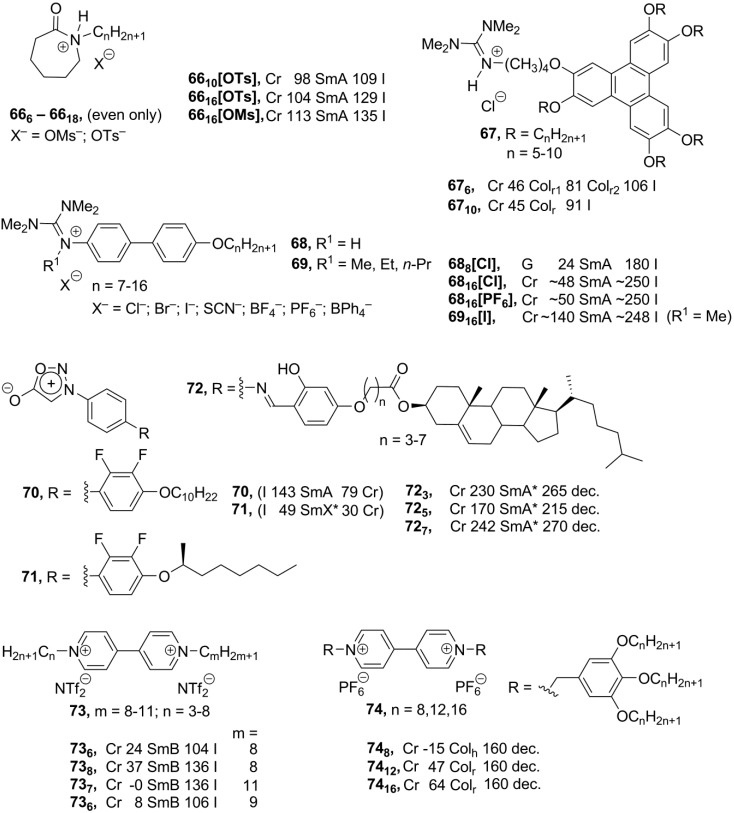


Above, we have considered ionic compounds, formed from classical ionic organic mesogenic groups: ammonium, phosphonium, imidazolium and pyridinium. Despite a huge variety of designed structures, the choice of the ionic organic mesogenic core is still rather limited. In order to overcome this problem, research toward the discovery of new applicable inexpensive ionic mesogenic units is highly important. Recently, a new family of liquid crystalline salts **66** (Scheme 16), based on the alkyl-substituted caprolactam cation, has been reported [91]. Caprolactam is available in a large scale from industry and reacts easily with alkylating sulfate reagents. The obtained caprolactam cationic compounds **66** (Scheme 16), substituted with long-chain alkyl groups, exhibited enantiotropic mesomorphism with the formation of smectic A phases, unfortunately only in very narrow temperature intervals. 

The combination of the triphenylene core and new ionic mesogenic moiety—guanidinium group leads to rectangular columnar enantiotropic liquid crystalline phases **67** (Scheme 16), formed in a relatively broad temperature range between 42 °C and 115 °C (for comparison, see [14,73,74,75,76,77,90]). Temperature- and chain-length-depended transitions between a common Col_r_ phase with *C/2m* symmetry and a new *P2m* symmetric Col_r_ mesophase have been observed within these new systems [92]. The calamitic guanidinium-based biphenyl ionic mesogens **68** form bilayered smectic A mesophases with hydrogen bonding between the guanidinium cation and counteranion. Stability of the mesophase and the temperature interval of the liquid crystalline state depend more on the employed counteranion than on the length of the *p*-alkoxy substituent in the biphenyl unit [93]. Detailed studies displayed that the counteranion is positioned along the N-H axes of the guanidinium cation and has pronounced hydrogen-bonding interactions with the N-H group of the cation. As a result, large counteranions disturb anisotropy of the molecule and destabilize the liquid crystalline phase. In the alkylated guanidinium compounds **69**, in which hydrogen bonding is no longer possible, stability of the mesophase is reduced severely [94]. The highly polarized and yet overall neutral character of zwitterionic sydnone systems **70–72** (Scheme 16) reveal them as promising structures for ionic chiral liquid crystals. Bearing chiral cholesteryl groups, salts **72** exhibited chiral smectic A mesophases. Although detailed structural investigations were prevented, due to unsatisfactory thermal behavior of the compounds **71**, a partially bilayered phase with either SmC* or hexatic structures was preliminary suggested [95]. 

As an alternative to the pyridinium charged core, the viologen-based compounds have lately been employed for the construction of ionic mesogens. It has been found that the symmetrically substituted viologen salts **73** (Scheme 16) with sufficiently long alkyl chains exhibit smectic B phases over a wide range of temperatures (from 0 °C to 140 °C). More specially, with slightly induced asymmetry in the molecular structure, the solid–LC transition temperatures could be lowered, and liquid crystalline properties of compounds **73** can be tuned by changes in the alkyl side-chains, attached to the viologen core [96]. In the further development, the complex viologen-derived discotic molecules **74** (Scheme 16) have been synthesized. They exhibit rectangular or hexagonal columnar phases, which are stable up to 160 °C. Additionally, redox behavior of salts **74** has been investigated [97].

The search for new unexpensive, stable, but yet easily chemically convertible cationic mesogenic groups is essentially important for the preparation of new mesogens possessing practically applicable properties. While the caprolactam-based systems did not form stable mesophases, the guanidinium- or viologen-derived salts appeared to be promising candidates for practically usefull mesogens. Viologen compounds could provide, for example, redox liquid crystalline systems (see also below). Guanidinium-based mesogens formed stable mesophases in wide temperature intervals, which could be expanded further by the right choice of counteranion and suitable cationic substitution pattern. 

## 7. Metallomesogens

**Scheme 17:**
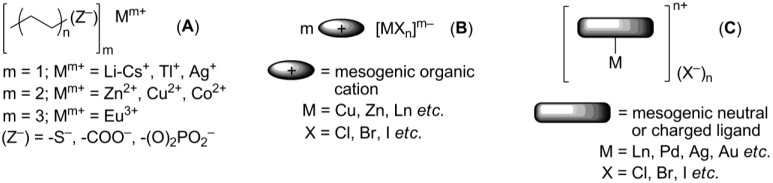


Ionic metallomesogens are metal complexes bearing ionic groups and displaying liquid crystalline properties. The incorporation of a metal center gives a great possibility to combine variable physical and physico-chemical properties of metal complexes with anisotropy of the aligned liquid crystalline arrangement [98]. This makes metallomesogens (both, neutral and ionic) attractive materials for broad practical applications, particularly as anisotropic magnetic liquid crystals [99], redox systems [100], luminescent liquid crystals for OLEDs [101] or solar sensitizers [102]. In this chapter, we focus mainly on the mesomorphic properties of ionic metal complexes. The possible and practical applications of metal-containing ionic crystalline materials will be discussed in a separate chapter below. There are several ways to introduce a metal center into the potentially mesogenic molecule [11]. In the simplest approach, positively charged metal particles, typically alkali or monovalent heavy metal cations, are combined with mesogenic “ate”-anions (usually, phosphates and carboxylates), bearing long hydrophobic hydrocarbon tails (Structure **A**, Scheme 17). Conversely, mesogenic large organic cations could form mesophases together with negatively charged metal-containing anions (Structure **B**, Scheme 17). Few examples, the uranium and europium-containing mesogenic salts **5_8_-5_20_**, **6[Eu(tta)_4_]** and **7[Eu(tta)_4_]**, **18[X]** (X = [EuBr_6_]^3–^Br^–^) have been already discussed above. Alternatively, a metal center could be connected with a multidentate mesogenic ligand to form a large complex cation, leaving small counteranions on the outer coordination sphere (Structure **C**, Scheme 17).

Mesogenic “ate” metal salts (**A**, Scheme 17) have been known for a long time [103]. They tend to form lyotropic mesophases (soaps) in the presence of solvents. Nevertheless, in the absence of a solvent they also exhibit thermotropic liquid crystalline forms. Recently, in a continuation of early studies [11], anisotropic electroconductivity of Na hexanoate (**A**, M^m+^ = Na^+^, (Z^–^) = -COO^−^, Scheme 17) and Co decanoate (**A**, M^m+^ = Co^2+^, (Z^–^) = -COO^−^, Scheme 17) have been measured [104]. It has been observed that due to a smectic anisotropic molecular arrangement, electric current conductivity along the cation-anion layers is larger by four orders of magnitude than that in the perpendicular direction. Tl pentanoate (**A**, M^m+^ = Tl^+^, (Z^−^) = -COO^−^, Scheme 17) exhibits a smectic mesophase in a range of 180–214 °C, which could be stabilized by the addition of Li pentanoate [105]. Long-chain Eu alkanoates (**A**, (Z^−^) = -COO^−^, n = 6–9, Scheme 17) display lamellar structures, where Eu ionic layers are separated by perpendicularly oriented alkanoate chains arranged in a hexagonal packing [106]. Unfortunately, the characteristic emission of Eu^3+^ is weakened by chelating carboxylate groups. The combination of the K^+^ cation and cholesteryl-derived sulfate anion gives a SmA mesophase in a temperature range of 139–238 °C [107].

Mesomorphism of the type **B** ionic metallomesogens (Scheme 17) is defined by the nature and size of mesogenic cation. These compounds find their application as luminescent molecules or precursors for inorganic nanoparticles (see below). For the latter purpose, the pyridinium-based salts **53_12_ [CuCl_4_]** (see structures **53[X]** in Scheme 14) have been synthesized [108]. It shows a smectic mesophase between 50 and 76 °C. Electrolysis of imidazolium liquid crystals **22[X]** (X^−^ = Ag(CN)_2_^−^ or Au(CN)_2_^−^, see structures **22[X]** in Scheme 10) produced Ag and Au nanoparticles, respectively [109]. These Ag and Au salts **22[Ag(CN)_2_]** and **22[Au(CN)_2_]** exhibit smectic A liquid crystalline phases, started around 60 °C. The silver complex **22[Ag(CN)_2_]** forms more stable mesophase than the gold analog **22[Au(CN)_2_]** (clearing points for **22[Ag(CN)_2_]** and **22[Au(CN)_2_]** are 157 °C and 112 °C, respectively). The imidazolium salt [C_12_mim]_3_[DyBr_6_] exhibits a smectic A liquid crystalline molecular arrangement at subambient temperatures and luminescence, characteristic for lanthanide complexes [110]. Between 127 °C and 224 °C, a smectic A mesophase has been observed for the pyridinium based salts bearing ZnCl_4_^2–^ anion [111]. Employed as a mesogenic group, the β-diketone moiety, bearing an alkoxyphenyl substituent, was attached to the *o*-position of the pyridinium ring.

**Scheme 18:**
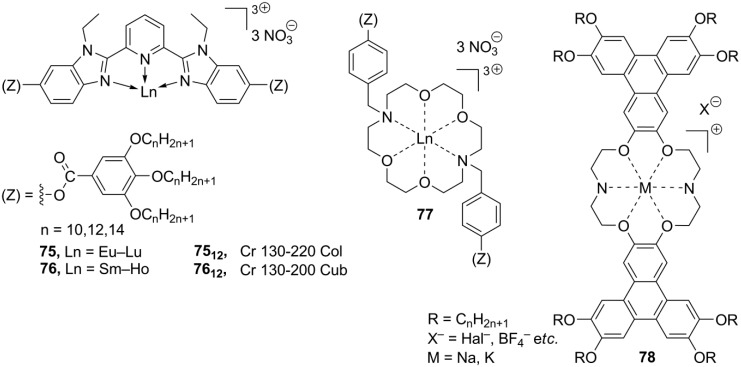


Together with structures **B**, considerable attention has been given to synthesis and studies of ionic metal complexes bearing a mesogenic ligand (**C**, Scheme 17). Recently, both types of metallomesogens, containing imidazolium groups, have been reviewed [112]. In the last decade, significant efforts have been directed toward synthesis of lanthanide mesogenic complexes, which combine luminescence with anisotropy in physico-chemical properties, induced by liquid crystalline molecular order. For most of so far synthesized lanthanide mesogenic complexes, counteranions (monodentate Cl^−^ or bidentate NO_3_^−^ anions) are found in the inner coordination sphere connected with the metal center [113,114]. Consequently, these compounds cannot be considered as true ionic mesogens with the complete separation of positive and negative charged species. For this reason, we give here only a general overview. The design of transition metal-based, especially lanthanide, mesogenic complexes is not easy [115]. Typically, a simple incorporation of a lanthanide metal center with the extended coordination core into a mesogenic ligand framework leads to molecules that do not form liquid crystalline phases [98]. In order to overcome this problem and preserve the mesomorphic properties after coordination with metal, several strategies have been developed. Those include a wrapping of the metal atom into a “cocoon” of aromatic rings, a connection of the ligand core with a large amount of flexible long-alkyl chains, an incorporation of rigid aromatic subunits in a periphery of the metal-ligand bulky core (“spatial ligand decoupling”) [114]. On account of these structural requirements and restrains, bulky ligands based on a bis(benzimidazole)pyridine core **75–76** (Scheme 18) or substituted Shiff base structures have been designed [112,113,114,115,116]. Other mesogenic lanthanide complexes have been obtained via the complexation of lanthanide salts with crown-ether derivatives (**77**, Scheme 18). They exhibited hexagonal columnar mesophases at temperatures above 60 °C [117]. In a similar approach, the liquid crystalline state could be generated in crown ether structures via incorporation into the ligand cavity of alkali metal cations [118,119]. For the crown-ether-derived ligands, bearing rigid lateral aryl or terphenylene subunits (**78**, Scheme 18), the influence of the alkali metal cation, counteranion and mesogenic substituents around the ligand-metal core on the structure of liquid crystalline phases and their stability at diverse temperature conditions have been investigated [120,121,122,123,124].

**Scheme 19:**
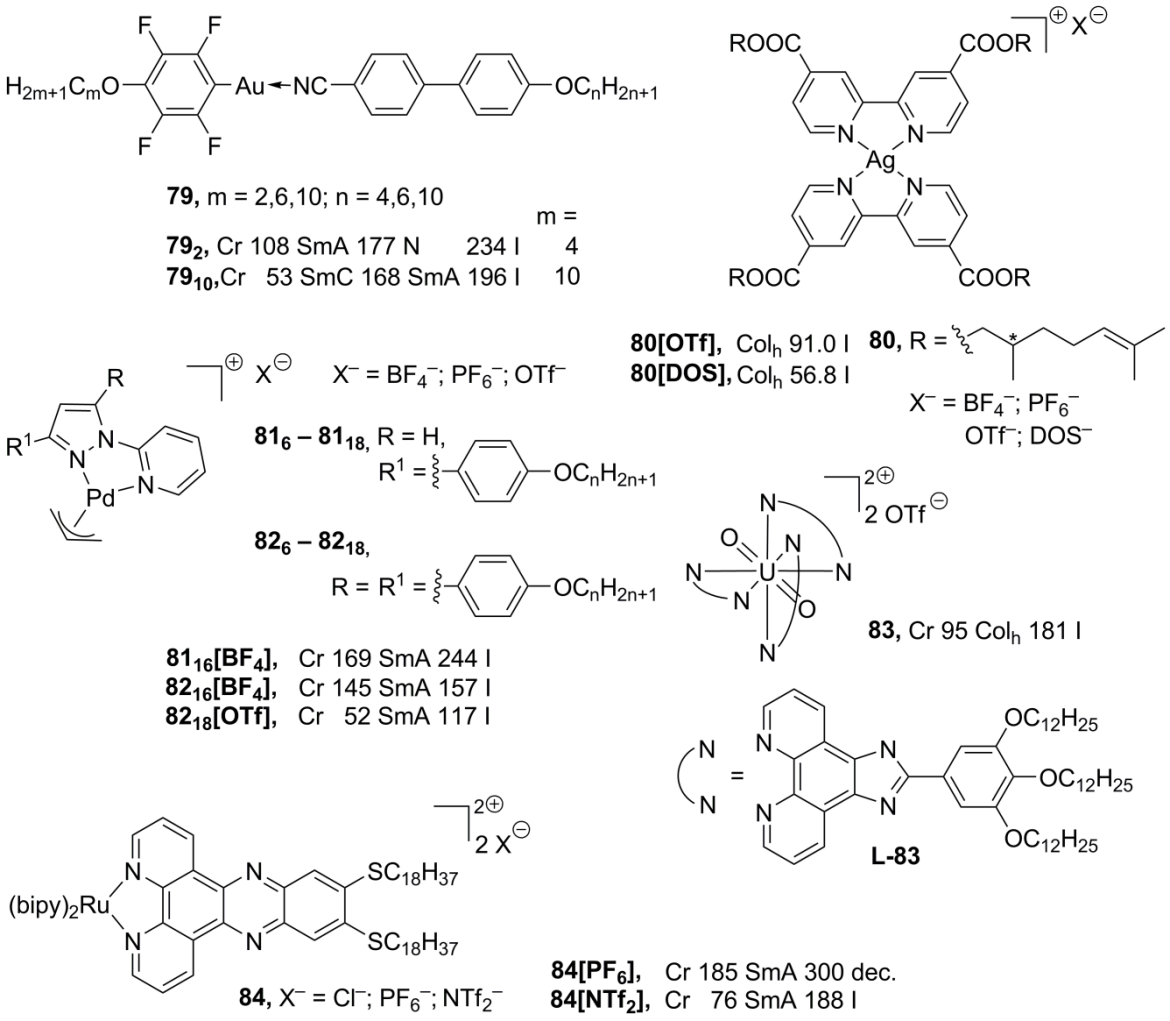


The rod-like geometry of gold(I) complexes favors the formation of lamellar mesophases. In addition, it is believed that weak intermolecular Au···Au interactions stabilize molecular liquid crystalline molecular organization [125]. Moreover, luminescence of gold linear complexes is attributed to weak intermolecular Au···Au contacts [126]. The structures **79** (Scheme 19), combining liquid crystalline properties and luminescence, have been recently reported [127]. These compounds exhibit rich mesomorphism: nematic, smectic A and C mesophases have been observed, depending on the substitution in the ligand. Surprisingly, Au···Au contacts have not been found in the solid state, and most possibly are absent in the liquid crystalline phase. Instead, the complexes **79** display very short intermolecular F···F interactions, which could be a driving force for a stabilization of the mesophase [127]. Coordination of silver with bipyridyl-derived ligands leads to the planar mesogenic silver complexes (**80**, Scheme 19). These structures **80** form hexagonal columnar mesophases, when OTf^–^ or DOS^–^ ions are employed as counteranions [128]. New bipyridino-derived silver mono- and polynuclear mesomorphic complexes have been recently prepared [129]. It has been observed that in the presence of a strongly coordinated anion (like NO_3_^–^ or saccharinate) these silver complexes do not form polymeric structures and do not possess liquid crystalline properties. Conversily, in the case of weakly or noncordinated counteranions (OTf^–^ or ClO_4_^–^) a lamellar-columnar mesophase is induced upon complexation with silver(I) [129].

Palladium metallomesogens **81**, **82** (Scheme 19), with a planar arrangement of ligands around the metal center, have been prepared from the substituted pyrazolylpyridine ligand precursors. The presence of a positive charge on the palladium-ligand core together with long lateral side-chain substituents is essential that the complexes **81**, **82** show mesomorphic properties [130,131]. The neutral dichloropalladium complexes melt directly into isotropic liquids around 170 °C, while the charged allylpalladium species form SmA phases with an interdigitaled bilayering organization. The Pd mesogens **82_18_[OTf]**, with two lateral long-chain substituents and the OTf^–^ counteranion, exhibit liquid crystalline phases at subambient temperatures, in the range of 50–90 °C [130], but the monosubstituted complexes **81** form smectic mesophases at relatively harsh conditions above 150 °C [131]. 

A new propeller-like design for metallomesogens with an octahedral configuration around the metal center has recently been presented [132]. The ionic complex **83** (as mixture of Λ and Δ isomers), in which a charged uranyl moiety was surrounded by three imidazolphenanthroline-based ligands, displayed a hexagonal columnar mesophase between 95 °C and 181 °C. In order to keep efficient packing order within the mesophase of **83** (Scheme 19), few consecutive stacking enantiomers should randomize a pile of molecules with an identical absolute configuration around the metal center, stacked on top of each other into polar aggregates. In general, most stable mesophases with imidazolphenanthroline ligands are shown by nonplanar lanthanide and uranyl complexes, with a ratio between the metal and ligand as 2:1 or 3:1 [133]. The phenanthroline-derived framework have also been employed for the design of new class of metallomesogens, highly luminescent (bipy)_2_Ru complexes **84** [134]. Regardless of the counteranion, the most of the complexes displayed smectic A mesophases at quite harsh temperatures, with an exception of the NTf_2_^−^ derivative **84[NTf_2_]**, forming a SmA phase already at 76 °C (Scheme 19).

Metallomesogens are very attractive compounds with regard to various practical applications. However, the presence in mesogen of a three-dimensional metal-ligand complex disturbs in most cases the stability of the mesophase. Conversely, in-plane coordinated metal centers stabilize the mesophase. Metals, such as palladium, silver or gold, are an excellent choice. They usually form planar complexes with T-shaped or square coordination of the metal center. In addition, in silver and gold complexes, weak argentophilic interactions or Au···Au contacts help to retain stability of liquid crystalline order. Consequently, these complexes exhibit liquid crystalline properties over a wide temperature range. In order to create a stable mesophase from nonplanar trasition metal complexes, a new “cocoon” synthetic strategy has been developed. By this new synthetic approach, it seems possible to get access to a variety of mesogenic three-dimensional metal complexes, which could find applications in different areas, from catalysis to optical electronics.

## 8. Self-Assembled Ionic Liquid Crystalline Systems

In the conventional design of an ionic liquid crystalline moiety, an ionic core is connected with mesogenic groups via chemical covalent bonding [11]. Alternatively, in ionic compounds, strong electrostatic interactions between a cation and counteranion can be used to build up liquid crystalline order on a supramolecular level [135,136]. By the connection of two or more structurally relatively simple charged particles via noncovalent forces, the tedious synthetic work could be avoided. By this general approach, called ionic self-assembly (ISA), a variety of attractive interactions, such as electrostatic contacts, hydrogen bonding, π-π interactions and van der Waals forces, has been employed for connecting of positively and negatively charged mesogenic cores together and creating a highly organized ionic liquid phase with supramolecular order [137]. All these interactions differ by strength, species or groups involved. Van-der-Waals and “amphiphilic” interactions are weak (up to 51 kJ·mol^−1^), non-directional, non-selective and operate on a short range. H-bonding is also rather weak interaction (up to 65 kJ·mol^−1^), but directional and selective [135]. The H-bonding interactions have been successfully used to create dimeric mesomeric structures (benzoic acids) or self-assembling multi-component systems [138,139]. In these structures, however, the problem of molecular recognition arises. The presence of complementary functional groups is required, which limites the choice of suitable molecules. The electrostatic forces are rather strong (up to 250 kJ·mol^−1^), but non-selective and non-directional. In this respect, a wide range of substrates can be used for the formation of ionic self-assemblies; the only requirements are the presence of cationic and anionic groups. However, the ionic self-assembly mechanism differs from the simple coulombic formation of salts. It has cooperative nature and, usually, includes the combination of many kinds of interactions. The cooperative character means that first bonds stimulate further binding, which propagates towards the final self-assembled structures [135]. In the last few years, the amount of published contributions in this subject increased dramatically with the purpose to build up the structures suitable for practical applications [140]. It was already described above that imidazolium based discotic molecules of substituted polyphenol ethers **37**–**39** (Scheme 11) could create supramolecular liquid crystalline phases via ionic self-assembly [10,71,72]. Recently, a new analogous system has been designed, in which an imidazolium unit is connected with polyphenols by a L-glutamic acid link [141]. When Br^−^ is used as a counteranion, after an initial columnar liquid crystalline molecular arrangement the formation of a cubic mesophase has been observed. 

**Scheme 20:**
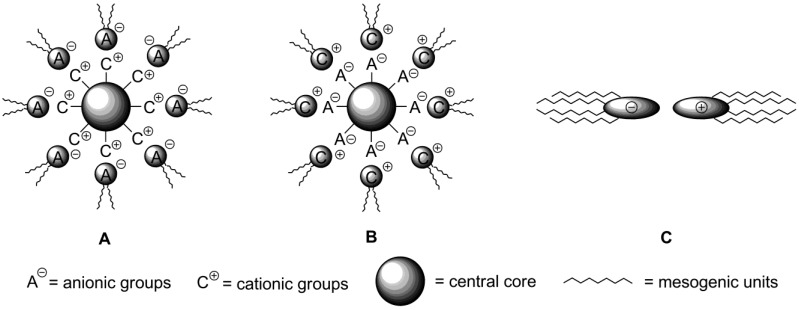


**Scheme 21:**
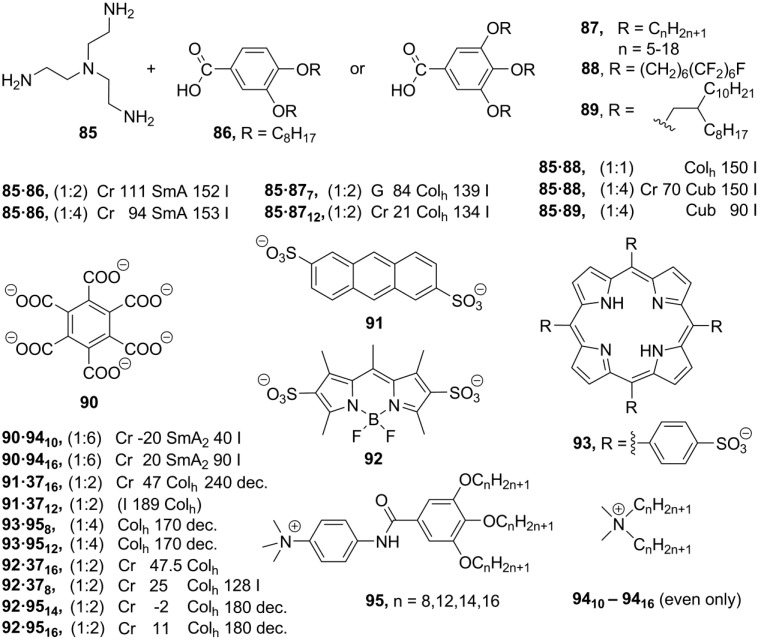


All self-assembled mesogens could basically be classified within three structural types. Structures **A** (Scheme 20) are constructed from a small sized discotic multicharged cationic core bounded by electrostatic forces with mesogenic anionic species. Conversely, the central core can be negatively charged and surrounded by mesogenic cations (**B**, Scheme 20). Alternatively, both cationic and anionic species may contain mesogenic units, then simple cationic-anionic liquid crystalline pairs can be build up (**C**, Scheme 20). The systems of **A**-type (Scheme 20) are usually designed starting from a branched flexible polyamine core, which is further protonated *in situ* by mixing with mesogenic polyphenol-based benzoic acids [141]. The counterparts form different liquid crystalline phases, depending on the ratio of the amine **85** and used benzoic acids **86–89** (Scheme 21). When compounds **85** and **86** are mixed in a ratio of 1:2, SmA mesophases have been observed, while hexagonal columnar molecular liquid crystalline order was obtained at a ratio between **85** and **86** = 1:4. Mixtures of **85** and **87** also exhibit Col_h_ phases. The melting and clearing temperatures can be tuned by changing the length of alkoxy-groups in polyphenolbenzoate moieties [142,143]. Micellar cubic mesophases with *Pm3n* symmetry have been obtained when branched or partially fluorinated alkoxy-substituents have been attached to anionic units, derived from acids **88** and **89** [144]. 

In order to build up the structures **B** (Scheme 20), the rigid aromatic carboxylate polyanions are used as the central negatively charged cores (e.g., see structures **90**–**93**, Scheme 21). Ammonium or imidazolium salts are typically employed as cationic counterparts (e.g., see structures **94**, **95**, Scheme 21). The benzenehexacarboxylate **90** was mixed with six equivalents of the ammonium cations **94** with variable alkyl chain lengths [145]. Surprisingly, no self-assembled columnar molecular arrangement has been observed. Instead, the mixture of **90** and **94** exhibited a bilayered smectic A_2_ mesophase at temperatures, started from −20 °C. In this molecular arrangement, the molecules of carboxylate-anion **90** are ordered with their planes parallel to layers. Within the second layer, alkyl chains from ammonium counterparts **94** are oriented perpendicular to the layer planes [145]. 

The self-assembled structures, based on the anionic luminescent disulfatoanthracene core **91**, have been constructed in a similar way. As cationic mesogens, ammonium and imidazolium-derived units **37**, **94**, **95** (Scheme 21) have been employed. Whereas ammonium-anthracenedisulfate systems did not form liquid crystalline phases, imidazolium-anthracenedisulfate mixtures displayed hexagonal columnar phases [146]. The ionic self-assembly method is an elegant solution for synthesis of luminescent ionic liquid crystals. Besides anthracenedisulfate **91**, the BODIPY- (boron-dipyrromethene) and porphyrine-derived anions have been applied as luminescent anionic cores for the preparation of self-assembled liquid crystalline structures. The BODIPY-modified fluorophore **92** (Scheme 21), bearing the polyalkoxybenzyl-substituted imidazolium cations **37** (in 1:2 ratio), exhibit hexagonal columnar fluorescent mesophases. In a solid state (and in a mesophase) emission is strongly red-shifted and broadened, showing a considerable degree of aggregation [147].

Alternatively, ammonium counterparts **95** have been mixed with BODIPY- and porphyrine-modified fluorophores **92** and **93** (Scheme 21). The mixtures build up hexagonal columnar mesophases, which are stable over a large temperature interval (from room temperature to 180 °C). It has been observed that the amido subunits in the ammonium parts are arranged in a hydrogen-bonded supramolecular network, stabilizing the thermotropic mesophase **[148]**.

**Scheme 22:**
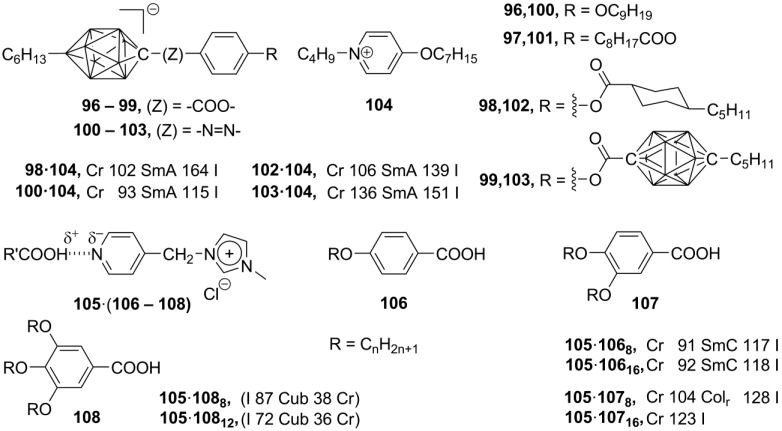


The simple combination of the [*closo*-1-CB_9_H_10_]^–^ carborane-anions **96–103**, substituted by mesogenic benzoate or azobenzene groups, with the heptyloxy-substituted *N*-butylpyridinium unit **104** provided partly interdigitated monolayer SmA mesophases (**C**, Scheme 20 and Scheme 22) [149]. Instead of electrostatic interactions keeping charged counterparts together, the hydrogen bonding can stabilize an ionic self-assembled supramolecular organization. Recently, it has been shown that, depending on the amount of alkoxy groups in compounds **106**–**108**, different mesophases can be created by mixing of pyridine-substituted imidazolium salts **105** and polyalkoxybenzoic acids **106**–**108** (Scheme 22). The imidazolium part served as H-bonding acceptor, while benzoic acids were used as H-bonding donor molecules. The *p*-monoalkoxybenzoic acids **106** together with the salt **105** form smectic C phases, while mixtures with *p,m*-dialkoxybenzoic acids **107** exhibit rectangular columnar phases. Finally, the treatment of **105** with *p,m,m*-trialkoxybenzoic acids **108** led to a cubic liquid crystalline phase [150].

By the ionic self-assembly (ISA) approach, a wide range of ionic liquid crystalline phases, having a complex structure and including different cores, e.g., the bio-compatible BODIPY fluorescent framework, can be easily obtained, avoiding tedious synthetic work. Usually, the assembly of ionic species occurs under a cooperative binding mechanism. The highly ordered complex mesophases with columnar or continuous cubic geometries, which could be potentially applicable for the construction of anisotropic ionic conductive materials, are formed by the ISA-method from simple components. In this respect, the consideration and use of other binding forces, such as e.g., “fit interactions” or H-bonding, together with electrostatic interactions could give new perspectives and further development of the ISA-synthetic approach [150]. 

## 9. Ionic Polymers and Dendrimers

**Scheme 23:**
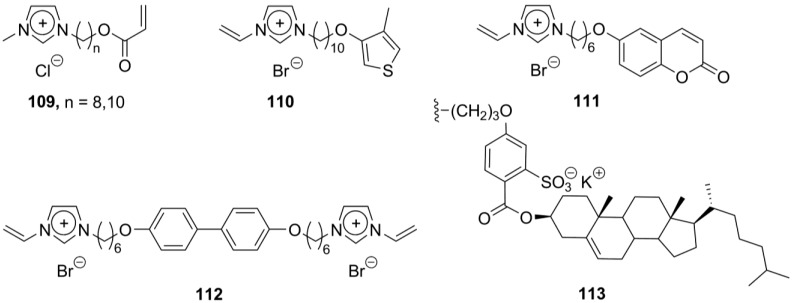


Incorporation of charged mesogenic groups into a polymer chain provides a new class of ionic liquid crystals, ionic polymer mesogens. In the presence of charged highly polarized ionic mesogenic groups attached to a polymer chain, the ion-ion electrostatic interactions become an important factor affecting the morphology of the polymer and supramolecular organization of the charged polymer species [151]. Ionic mesogenic substituents are able to provide tight contacts with solvent molecules (especially with highly polarized protic and aprotic solvents) or allow an ionic self-assembly of charged polymer chains into highly organized microdomains [152]. Consequently, ionic polymers can easily form lyotropic mesophases in the presence of solvent molecules or thermotropic liquid crystals in the absence of a solvent. Since the ionic polymers have been already reviewed by Binnemans in 2005 [11], we mainly focus here on the recent developments during the last five years. Due to easy synthesis and variability, imidazolium-mediated mesogens have widely been employed for the design of ionic polymers. The conventional design of imidazolium-based monomers (Structure **A**, Scheme 6) has been modified by variations of alkyl substituents and the incorporation of polymerizable acrylate groups (**109**, Scheme 23). The lyotropic mesomorphism of the imidazolium monomers and 3D bicontinuous cubic lamellar phases, created upon polymerization, have been investigated [153]. The polymerization of the vinylimidazolium salts having a thiophene group, attached via a flexible alkyl spacer (**110**, Scheme 23), gave electrochromic polymer films consisting of liquid crystalline domains. The detailed investigation revealed that the polymerization occurred on both thiophene and vinyl sides. In the lamellar supramolecular structure, the sheets of thiophene polymer are oriented perpendicular to the polymerized vinylimidazolium networks [154]. The vinylimidazolium monomers **111** and **112** (Scheme 23), bearing coumarin groups or connected to the p,p’-dioxy-biphenyl core, respectively, were polymerized in the presence of the AIBN initiator. The monomer **112** gave the polymer, exhibiting a liquid crystalline phase above 127 °C (no information given about the structure and clearing point) [155]. In an alternative approach, negatively charged sulfate groups were used to transform nonpolar hydrophobic hydrocarbon polymer chains into ionic polymer structures. The cholesteryl-based neutral ester and anionic sulfate units (**113**, Scheme 23) have been attached to the polysiloxane backbone via a Pt-mediated graft polymerization.

The incorporation of a photosensitive azobenzene unit [69] into an ionic polymer chain provided photoactive ionic liquid crystalline polymers. The polymers showed smectic and cholesteric molecular arrangements in the liquid crystalline state at temperatures above 66 °C [156]. A cross-linked polymerization of polysiloxane and calamitic azobenzene molecules (derived from “brilliant yellow”) resulted in liquid crystalline elastomers, displaying smectic molecular order upon heating to 180 °C [157]. The supramolecular material with photoinduced anisotropy properties was obtained viaionic self-assembly of small negatively charged *p*-(*N,N*-dimethyl)-azobenzene sulfate units to a hydrocarbon polymer chain with positively charged pyridinium side-substituents. These supramolecular assemblies build up lamellar layered structures, and after irradiation with an UV laser show a very pronounced anisotropic orientation [158]. Alternatively, long azobenzene-derived rod-like ammonium cations have been assembled with a poly(acrylic) acid polymer containing free carboxylate groups. After irradiation with a laser, azobenzene chromophores have been observed to become oriented in the direction perpendicular to the laser polarization [159]. The poly(methacrylic) acid-based polymers with piperidinium azobenzene units as end groups exhibit smectic A phases in a range of 35–155 °C. This temperature interval is much higher than that observed for nonionic analogs, and this was attributed to the ionic aggregation in the charged polymer [160]. 

**Scheme 24:**
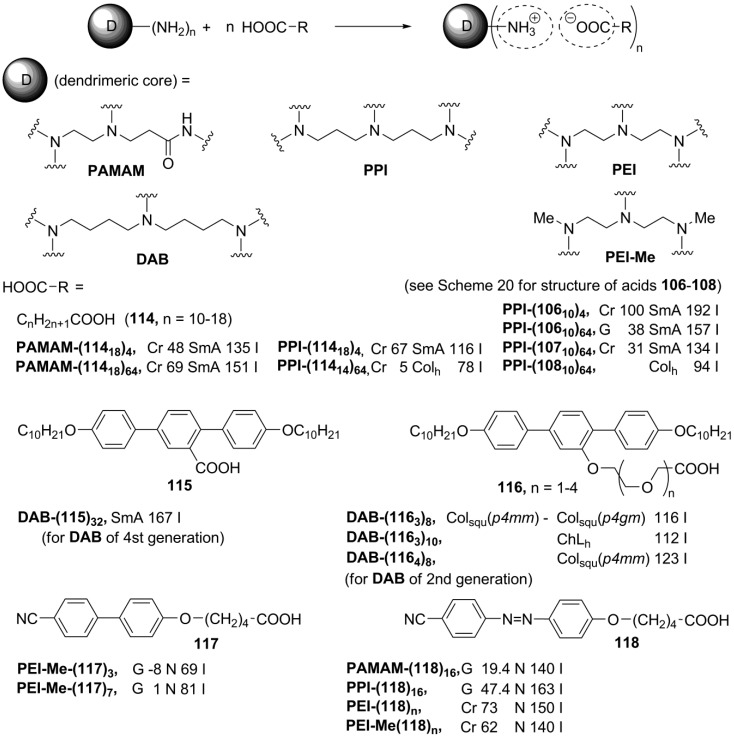


The conventional design of dendrimer liquid crystals includes a central dendrimer core linked to periphery mesogenic groups via a hydrocarbon spacer. The formation of a LC phase is a result of the molecular arrangement at the supramolecular level, which is determined by the microsegregation of the three molecular regions with very diverse characteristics: a central dendrimer core, mesogenic units and terminal flexible chains [161]. In ionic dendrimer mesogens, liquid crystalline order arises from the segregation of charged groups in molecules, therefore, in this case, the presence of mesogenic groups is not crucial. The typical way to generate the ionic dendrimer molecules is to modify the basic amino groups on a periphery of a polyamino dendrimeric core by the reaction with long-chain carboxylic acids. By this approach, PAMAM- (polyamidoamine) and PPI-based (polypropyleneimine) ionic mesogenic dendrimers have recently been synthesized (**114**, Scheme 24).

These systems exhibit smectic A phases, except for the PPI-derived dendrimers of 5th generation, forming columnar self-assemblies [162]. When polyalkoxysubstituted acids **106**–**108** (Scheme 22) have been used for the protonation of the PPI-dendrimeric substrate, the formation of smectic (with **106** and **107**), hexagonal and rectangular columnar (with **108**) phases has been observed [163]. It was later discovered that these dendrimer molecules are luminescent in solution and these emission properties were retained in the liquid crystalline state [164].

The *in situ* protonation of diaminobutane-derived (DAB) dendrimeric cores (1st–5th generations) with facial amphiphilic carboxylic acids (**115** and **116**, Scheme 24) led to the mesogenic ionic self-assemblies. The mesophase structures are mainly depended upon a ratio of a dendrimer to carboxylic acid and the length of the lateral polymer spacer to carboxylate groups in counterparts **115** and **116**. The increased degree of the molecular organization in the mesophases, depending on the ratio of reagents and structure of the acid counterpart, was observed in a sequence: SmA, ChL_h_ (hexagonal channeled layer phase), Col_h_, Col_squ_ (*p4gm* symmetry), Col_squ_ (*p4mm* symmetry) [165]. 

Unexpectedly, the self-assembled ionic systems, based on PEI-Me (methylated polyethylenimine) dendrimers and *p*-cyanobiphenylpentanoic acid **117** (Scheme 24), displayed the formation of nematic phases. The temperature mesophase intervals (−12–80 °C) were moderately affected by changes in a ratio of the reagents [166]. The further modification of an acid counterpart provided photosensitive ionic dendrimers, forming nematic phases. The carboxylic acid **118**, containing an azobenzene moiety (Scheme 24), was assembled with PAMAM, PPI, PEI and PEI-Me based dendrimer cores. Stable birefringence was induced within the obtained nematic thermotropic systems using the linearly polarized light [167].

The physical properties, in particular the mesomorphic behavior of ionic polymers differ considerably from those revealed by small ionic mesogens. The formation of continuous polymer chains or networks allows creation of homeotropically aligned polymeric monodomains on a surface and, consequently, retains continuous liquid crystalline order along a mesophase. This is very important concerning, for example, 1D transport of ions. Alternatively, a construction of polymeric sheets from thiophene monomers can enhance hole conductivity in a liquid crystalline phase. This phenomena can be used for construction of hole conductors with organized liquid crystalline structure for OLEDs or molecular electronics.

The ionic self-assembly of dendrimeric molecules give complex structures with unique mesomorphic properties, in particular stable discotic nematic D phases. The incorporation of the azobenzene groups to these nematic phases is one perspective direction, which could lead to new photoactive materials with nematic liquid crystalline organization. 

## 10. Applications of Ionic Mesogens 

In this chapter, we give an overview on the actual trends in potential and practical applications of ionic mesogens. Ionic liquid crystals have the typical characteristics for ionic liquids, and, at the same time, an organized structure of a liquid crystalline phase. This leads to the unique combination of ionic conductivity and high polarizability, displayed by ionic liquids, and anisotropic physical and chemical properties, revealed by liquid crystalline materials [11]. Nowadays, these kinds of liquid crystals are expected to serve as anisotropic ion-conductors, ordered reaction media or templates for synthesis of zeolites, mesoporous materials and nanomaterials. Additionally, ILCs also possess a high potential as electrolytes in dye-sensitized solar cells.

Recently, it has been discovered that the reductive decomposition of pyridinium-based salts **53_12_ [CuCl_4_]**, bearing CuCl_4_^2–^ anion, led to the CuCl nanoplatelets with a well-developed crystal habit and a tunable particle size. In the original procedure, CuCl nanoparticles were obtained at elevated temperatures by the reaction of **53_12_ [CuCl_4_]** with the reducing agent, 6-O-palmitoyl ascorbic acid [168]. The morphology of CuCl nanoparticles was a result of the layered molecular arrangement in the liquid crystalline precursor. The particle size, thickness and connectivity can be tuned by varying the reaction temperature [169]. In detailed studies, following the original contribution, it has been observed that the reduction of the CuCl-precursor **53_12_[CuCl_4_]** started at temperatures far below 100 °C even in a solution phase, giving the inorganic CuCl macroporous network [108]. The two-component system of **53_12_[CuCl_4_]** and 6-O-palmitoyl ascorbic acid showed complex decomposition behavior [170], therefore further research was directed toward the preparation of single-component ionic precursors for inorganic particles. According to this methodology, electrolysis of the imidazolium liquid crystalline salts **22[Ag(CN)_2_]** and **22[Au(CN)_2_]** gave Ag and Au nanoparticles, respectively. The size of the particles was controlled by a variation of potential and current density [109]. Alternatively, gold nanoparticles have been obtained by photolysis of the imidazolium salt **53_12_[AuCl_4_]**. Upon mechanistic studies, it has been concluded that photoreduction of AuCl_4_^-^ anions leads to Au(0) atoms embedded into a liquid crystalline matrix of **53_12_[AuCl_4_]**. Depending on the viscosity of the system at different temperatures, either Au nanoplatelets or spherical particles can be produced [171]. Blue- and green-luminescent ZnO nanocrystals, supported with imidazolium mesogens bearing carboxylate group, have been obtained via hydrolysis of intermediate Zn-carboxylate products [172].

**Scheme 25:**
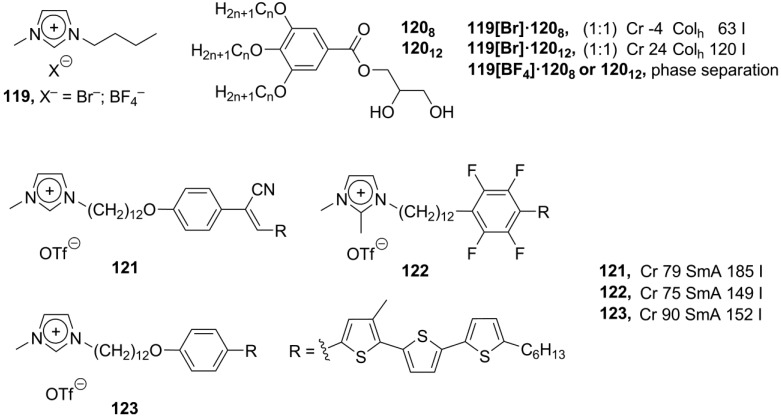


Due to an identical orientation of molecules in a mesophase (orientational order), liquid crystals possess anisotropic properties, in particular, anisotropic conductivity of charge carriers, such as holes, electrons or ions [137]. Ionic mesogens are consisted of cation-anionic pairs; therefore, ionic liquid crystals are most suitable materials to provide ionic anisotropic conductivity [173]. The ionic self-assembly approach opened a way to new ionic liquid crystalline supramolecular arrangements, with use as 1D or 2D ionic conductors [137]. Ion conductive mesophases of columnar or cubic morphology, which are most efficient for the ion mobility, have been constructed by a self-assembly of imidazolium and pyridinium-based mesogens [140]. Recently, new 1D ion conductive columnar mesophases have been prepared, utilizing a “noncovalent” supramolecular design. The mesogens, were assembled via noncovalent interactions from two components, ionic imidazolium salts [C_4_mim]^+^X^−^ **119** and polar multi-substituted diols **120** (Scheme 25). Depending on the ratio of components, the systems **119**·**120** exhibited columnar phases in the temperature range of 0–80 °C. Compared with the related imidazolium salts **37** and **38** (Scheme 11), the systems **119**·**120** showed highest conductivity along the columnar axes (*σ*_||_ = 3.9 × 10^−3^ S cm^−1^ for **119**·**120** *vs.* 10^−5^–10^−8^ S cm^−1^ for **37** and **38**), and displayed anisotropy of ionic conductivity (*σ*_||_ /*σ*﬩) in a range of 8–87, depending on the length of alkoxy-substituents [174].

Conductivity of neutral triphenylene mesogens have been compared with that provided by the ionic columnar and cubic mesophases, created by the compounds **45** and **46** (Scheme 12). The studies showed better characteristics for compounds **45** and **46** over their neutral analogs [77]. Interestingly, for the *L*-glutamic-based imidazolium salt with Br^–^ anion, ionic conductivity displayed some decrease upon the columnar-micellar cubic transition [141]. For a stabilization of the continuous liquid crystalline molecular organization in the homeotropically aligned mesophases, polymerazable substituents have been introduced into an ionic mesogenic core. After polymerization, stable ordered conductive molecular thin films have been obtained [175]. The imidazolium salts **39** (Scheme 11), bearing acrylate groups, create one-dimensional ion conductive polymeric films under photopolymerization conditions. Prior to the polymerization procedure, the self-assembled molecular columnar substructures of the mesophase can be homeotropically aligned either perpendicular to the substrate surface or, by shearing, with the parallel orientation. Anisotropy of conductivity (*σ*_||_ /*σ*﬩) reached 260–1400 for the polymerized samples at different temperatures [72]. In the new imidazolium-based salts **121–123**, reported recently, ionic conductivity was combined with an electronic charge transport by an incorporation of a trithiophyl subunit into an imidazolium mesogen (Scheme 25). The compounds **121–123** exhibited smectic A phase, in which high ionic conductivities were observed. In addition, these compounds displayed efficient electrochromism in the liquid crystalline bulk state [176]. The viologen-based mesogens **73** (Scheme 16) have been used to fabricate carbon composite electrodes, with the purpose to improve their characteristics [177]. 

**Scheme 26:**
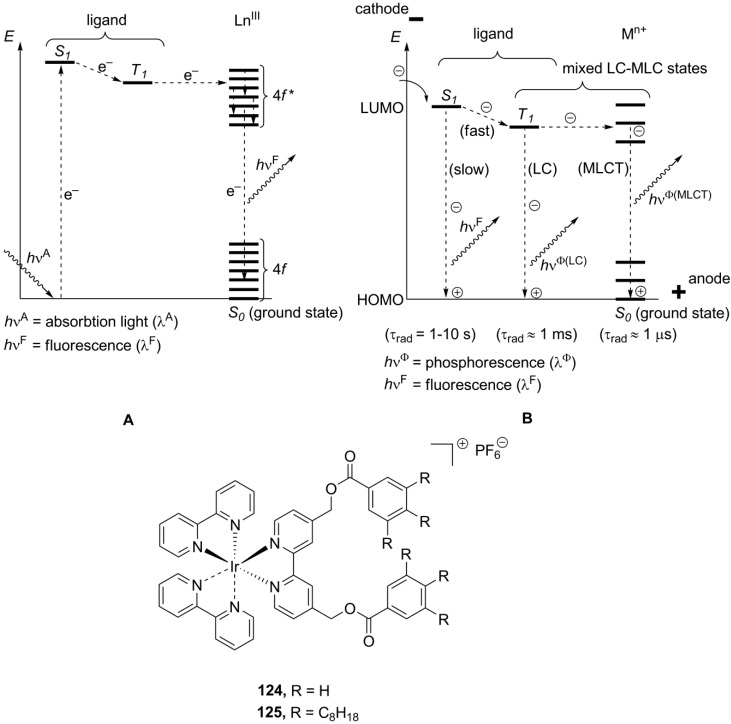
A schematic representation of luminescence process in lanthanide complexes **(A)** and electron-hole phosphorescent recombination in late-transition metal complexes (**B**).

The liquid crystalline materials easily and reversibly respond to electric and magnetic stimuli. These properties have successfully been applied for the construction of LCD displays. However, due to the use of the dichroic sheet polarizers and absorbing color filters, the viewing angles and brightness of LCD displays are rather insufficient. In order to omit additional polarizers and color filters and, consequently, optimize the optical parameters, the application of luminescent LCs as bright light emitters is highly desirable [178]. In this respect, lanthanide mesogenic complexes have been proposed as potential candidates for bright luminescent emitters [179]. Lanthanide metals are well known for their ability to display emission spectra with very narrow emission bands, giving the high purity color of the luminescent light [180]. Upon an excitation, electron transfers to an excited singlet state (*S_1_*) then to a triplet (*T_1_*) state of the ligand, and, finally, to an excited 4*f*-state of the metal center. Upon relaxation, the sharp spectroscopic bands originate from the *f*-*f* transitions within the metal center (**A**, Scheme 26) [179,181]. The ligand structure has a slight influence on the shift of the emission wavelength, but a pronounced effect on the molar extinction coefficient and, consequently, on the intensity of an emitted light [181]. A lanthanide-containing mesophase could easily be obtained by doping of the non-luminescent ionic liquid crystalline substrate with a photoactive lanthanide complex. The ionic liquid crystalline phase, formed from [C_12_mim]Cl (**A,** m = 1; n = 12; X^−^ = Cl^−^, Scheme 8) and 1 mol% of a phenanthroline-based lanthanide complex exhibited intense NIR (near IR) luminescence at room temperature [182]. The mesophase is stable in a range of 10–100 °C, and the intensity of the emitted light is enhanced in a liquid crystalline phase, compared with parent complexes in solid state or solution. As an example, the measured quantum yields of the Yb-containing material were 1.5 times higher than for the parent [(tta)_4_Yb(phenanthroline)] (2.1% *vs.*1.6% (as a solid) *vs.* 1.4% (as a toluene solution)) [182]. The synthesis of new ligands, based on a “cocoon” design (see above and Scheme 18, Scheme 19), made a preparation of the lanthanide mesogens possible, which exhibited liquid crystalline phases at room and subambient temperatures [113,114,115,116,117,133]. The lanthanide complexes **77** (Scheme 18), based on aza-crown-ether ligands, displayed columnar hexagonal mesophases in wide temperature windows and retain their luminescent properties almost up to the clearing point [117]. 

Lanthanide complexes with the phenanthroline-derived ligand **L-83** (Scheme 19) formed a luminescent cubic mesophases above 215 °C [133]. Unfortunately, the molar extinction coefficient of intraconfigurational *f-f* transitions within a lanthanide ion is rather low, usually less than 10 L·mol^−1^·cm^−1^, leading to weak luminescence of lanthanide complexes [183]. In this respect, parallel with the design of new lanthanide-based mesogens, the new liquid crystalline metal complexes have been investigated. The recently synthesized silver complexes **80** exhibited hexagonal columnar phases, consisted of the dimers with bridged triflate or DOS^–^ anions. These mesophases displayed unusual blue luminescence, probably due to excimers arising from argentophilic interactions in the columns of stacked dimeric structures [128]. 

Within the past decade, new highly efficient triplet emitters, based on late-transition metal complexes, have become highly attractive, in particular, due to their applicability in organic light-emitting diodes (OLEDs) [184]. Under an electrical excitation, both, singlet and triplet, excited states are generated in an emitter. In a conventional organic-based emitter, the singlet-triplet (*S_1_-T_1_*) transitions between excited states are spin-forbidden, and the fluorescence occurs entirely from a relaxation of an excited singlet state (*S_1_-S_0_*), giving low quantum yields (theoretical maximum is only 25%) [185]. In late-transition metal complexes, the *S_1_-T_1_* transitions are allowed by a spin-orbit coupling induced by the presence of the metal center. In this mechanism of the relaxation, electron transfers from an initial excited singlet state *S_1_* to a triplet state *T*_1_, located on the LUMO of the ligand, and then to the HOMO of the ligand (LC-transfer) or the metal center (MLCT-transfer), causing phosphorescence of the complex (**B**, Scheme 26) [186]. Due to a triplet harvesting phenomena, the theoretical quantum efficiency of phosphorescence in late-transition metal complexes reaches 100% [187]. Among a huge variety of studied metal complexes, derivatives of Ir, Os, Ru appeared to be most suitable for potential applications in OLEDs [184]. The alignment of the phosphor molecules in orientational order of a liquid crystalline phase could simplify the hole transport from an ITO anode to a doped emission layer (EML) of an OLED device. As a potential application, the ionic metallomesogenic phosphors could be used to fabricate emitting ordered liquid crystalline films. The high polarization of ionic molecules and high ion conductivity should enhance the hole transport, as well, consequently, an efficiency of an OLED device. Recently, new ionic Ru-based complexes **84** (Scheme 19) have been reported, exhibiting smectic A phases up from 76 °C. Bright luminescence of the complexes **90** has been observed, and the color of luminescence can be tuned by changes of the counteranion [134].

In an alternative approach, new tris-piridyl-based iridium complexes **124** and **125** bearing linked polyalkoxybenzoate moieties have been synthesized (Scheme 26) [188]. The iridium derivative **125**, substituted with C_8_-alkoxy groups, displayed liquid crystalline behavior and induced a columnar hexagonal mesophase by fast cooling of an isotropic liquid. The decrease of order on going from the crystalline to the frozen liquid-crystalline state in **125** is followed by a shift of the luminescence wavelength from green to yellow and only a slight decrease in the luminescence quantum yield. A further shift in the emission color to orange-red has been observed in formed by spin coating amorphous thin films. By heating these thin films, the solid crystalline order can be restored, giving back green luminescence, and this process is fully reversible [188].

**Scheme 27:**
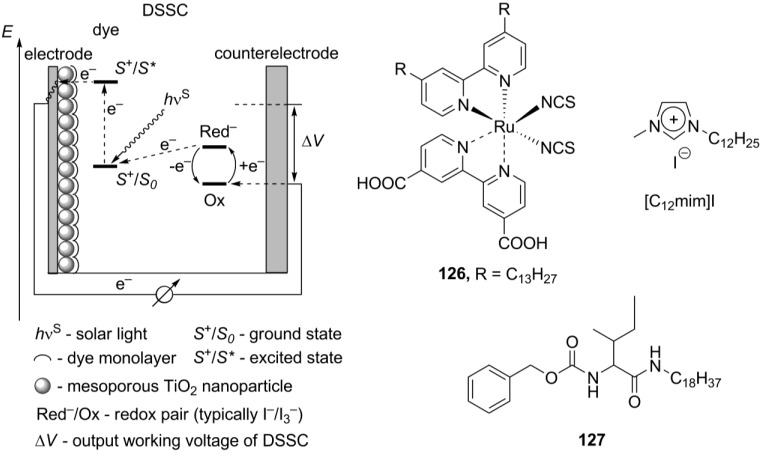
Schematic representation of a DSSC.

In 1991, a photovoltaic solar cell of new type (so-called dye-sensitized solar cell, DSSC), was reported [189]. The heart of DSSC is a thin mesoporous oxide layer, composed of TiO_2_ nanoparticles, sinthered together to establish electronic conduction. The oxide layer is placed on a transparent conducting oxide (electrode, Scheme 27) and covered on the surface by a monolayer of a charge-transfer dye. A solar photoexcitation causes an injection of electron to the conduction band of the oxide, leaving the dye in its oxidized state. The subsequent migration of electron to an electrode and further on via an electric chain to a counterelectrode generates electric current. The dye is restored to its ground state by a redox electron transfer from an electrolyte, containing a redox system, which is subsequently regenerated on the surface of a counterelectrode (Scheme 27). Since the transfer of electron from the redox system to the dye is much slower than an injection of electron to the TiO_2_ connection band upon the photoexcitation, the DSSC-based devices are able to generate an electric current with overall theoretical efficiency limit of 34%. Practically achieved efficiency of this type of solar cells has reached 12%. All aspects concerning DSSCs are discussed in detail in a very recent review [190]. One of the essential components of a dye-cell is an electrolyte. The function of an electrolyte is the transfer of charged components of a redox system from the counterelectrode to the oxidized dye molecule, thus regenerating the ground state of the dye [191]. Originally [189] and nowadays [190], electrolytes, based on organic solvents, e.g., acetonitrile, and containing ionic additives, give best output efficiencies. During the last years, ionic liquids have been considered as an environmentally friendly alternative to conventional organic electrolytes [191,192]. The rate of the ion transport is one of the factors, limiting the productivity of the dye-sensitized solar cell. The ion mobility and, consequently, electrical energy conversion efficiency of the cell can be improved considerably by a decrease of the viscosity of an electrolyte. For example, the diffusion coefficient *D* of I_3_^−^ anion in acetonitrile (viscosity 0.34 MPa s^−1^), used in record DSSCs, is 1.5 × 10^−5^ cm^2^ s^−1^, while *D* for [C_3_mim]I (viscosity 880 MPa s^−1^) amounts to 1.9 × 10^−7^ cm^2^ s^−1^ [190].

As discussed above, the ion conductivity of an ionic liquid electrolyte can be enhanced by assembling molecules into liquid crystalline order [137,140,173,174]. In this respect, new ionic liquid crystalline electrolytes, based on the imidazolium cation, have been synthesized [193]. The equimolar mixture of iodine and the imidazolium salt, [C_12_mim]I (**A,** m = 1; n = 12; X^−^ = I^−^, Scheme 8), exhibited a smectic A mesophase between 27 °C and 45 °C. The dye-sensitized solar cell with this mixture displayed maximum short-circuit photocurrent *J_sc_* around 7 mA cm^−2^, while the cell with the mixture of [C_11_mim]I/I_2_, which does not show liquid crystalline properties, gave *J_sc_* around 6 mA cm^−2^. The measured diffusion coefficients for both electrolytes were 4.2 × 10^−8^ cm^2^ s^−1^ and 3.2 × 10^−8^ cm^2^ s^−1^, respectively. Homeotropically aligned samples of [C_12_mim]I/I_2_ exhibited anisotropy in ion conductivity for the directions, parallel and perpendicular to the director vector *ñ*. It has been postulated that in the smectic A molecular arrangement the I^−^ and I_3_^−^ anions are moved between smectic A layers, thus promoting the redox reaction between the dye and I^-^ anion (Scheme 27) [193]. Later, it has been observed that the output characteristics of the DSSC, based on the amphiphilic Ru-dye **126** (Scheme 27), can be enhanced by the use of an ionic liquid crystalline mixture [C_12_mim]I/I_2_. Perhaps, the dye with C_13_ long chain substituents perfectly matches anisotropic orientation order of the ionic liquid crystalline electrolyte [194]. In addition, the maximum short-circuit photocurrent *J_sc_* has been increased by forming the quasi-solid-state ionic liquid crystal DSSC (based on N719 Ru-dye) in the presence of the low molecular weight gelator **127** (Scheme 27) [195]. The imidazolium electrolyte is also operational in the solid crystalline state, though fabricated “all-solid-sate” DSS cells displayed lower electrical energy conversion efficiency then those constructed with ionic liquid crystalline electrolyte (2.6% *vs.* 3.51% for DSSCs based on dye **126**) [196]. 

Ionic mesogens are also finding use in molecular biochemistry applications. As an example, new dialkoxy-substituted *N*-benzylimidazolium salts have been synthesized [197]. These compounds showed rich mesomorphism and formed smectic A (molecules with short alkyl tails) or hexagonal columnar phases (molecules with long chain substituents). The rare *Ia3d* bicontinuous cubic liquid crystalline phases have also been obtained and characterized. These ionic mesogenic molecules, when formulated with colipides, enhance the transport of active siRNA into a biological cell. The obtained results show a high transfection efficacy of formulated compounds and a high silencing efficiency with more than 80% inhibition of the targeted gene at 10 nM of siRNA concentration. Thus, the potency of amphiphilic imidazolium salts as a new generation of the RNA transport system has been demonstrated [197,198].

As it was demonstrated in this chapter, ionic liquid crystalline materials can find suitable applications in many areas of interest, from synthesis of inorganic nanoparticles to construction of OLEDs or solar cells of a new generation. One of the perspective directions could be an application of ionic-self assembled systems, possesing high anisotropic ion conductivity, as liquid crystalline electrolytes for fabrication of highly efficient dye-sensitized solar cells.

The late transition metallomesogens could be used for the preparation of highly ordered liquid crystalline emitting films for phosphorescent OLEDs. The self- or directed organization of metallomesogenic molecules into liquid crystalline orientational order could enhance hole transport directly to metal center and, consequently, the efficiency of a fabricated OLED device.

## 11. Summary and Outlook

The first thermotropic ionic liquid crystalline phases were obtained a long time ago. However, until recently, ionic mesogens have not received much attention. In the last two decades, the situation has changed dramatically. Due to the presence of charged particles in a mesophase, ionic liquid crystals reveal different mesomorphic behavior and phase morphology, as compared to neutral mesogens. The calamitic ionic molecules are constructed from a positively charged head and long hydrocarbon tails. This structure favors the formation of smectic lamellar phases via a combination of the electrostatic ion-ion interactions, stabilizing the structure of a layer, and Van-der Waals forces, organizing the arrangement of hydrophobic tails between ionic layers. Among a huge variety of so far obtained ionic mesogens, most of them are derived from the substituted cationic imidazolium framework, although ammonium, phosphonium, pyridinium and other charged mesogenic cores have also been employed for the design of ionic mesogenic molecules. In this field, new cationic mesogenic units, which could easily be accessed and further modified via simple synthetic transformations, are in high demand. As an illustration, the mesogens containing viologen or guanidinium-derived cationic groups displayed rich mesomorphism and form stable ionic mesophases. In an alternative synthetic approach, ionic self-assembly methodology (ISA) provides the possibility to build up ionic liquid crystalline complex structures from relatively simple charged components. The ionic self-assembled phases contain the highly branched network of channels, enhancing the ion transport and, consequently, ionic conductivity of a mesophase. Ionic mesogens are characterized by the ability to spontaneously create homeotropically aligned liquid crystalline phases with continuous orientational molecular order. The combination of enhanced ionic conductivity with liquid crystalline anisotropic properties makes ionic mesogens highly demanding materials for molecular electronics. Nowadays, the ionic liquid crystalline electrolytes are widely used for the fabrication of environmentally nonaggressive solar cells, having a reasonable electrical energy conversion efficiency and, at the same time, displaying a high efficiency over a very long operational period in harsh conditions, passing, for example, the stability test over 1000 h at 80 °C.

The synthesis of ionic metallomesogens is a highly challenging task, because the presence of a multicoordinated metal center typically disfavors the formation of a liquid crystalline phase. However, in the last years, a number of ionic mesogenic transition metal complexes, which are able to exhibit a mesophase at subambient temperatures, have been synthesized. This opens a way to the new luminescent and phosphorescent liquid crystalline materials possessing high ion conductivity. Potentially, the late transition metallomesogens could be employed for the preparation of highly effective phosphorescent OLEDs.

In conclusion, ionic liquid crystals display many interesting and useful features, among them, enhanced ion conductivity together with the ability to spontaneously create homeotropically aligned mesophases. Despite a huge variety of already synthesized ionic mesogenic systems, there is always room for further structural improvements, leading to interesting and variable mesomorphic properties, suitable for subsequent applications.
